# Cascade-activatable small-molecule theranostic nanomicelles for photodynamic-immunotherapy of immune-cold lung tumors

**DOI:** 10.1016/j.ajps.2026.101152

**Published:** 2026-04-29

**Authors:** Young-Chan Yoon, Hyoung-Jun Kim, Yongdoo Choi

**Affiliations:** Division of Technology Convergence, National Cancer Center, 323 Ilsan-ro, Goyang, Gyeonggi-Do 10408, Republic of Korea

**Keywords:** Activatable nanotheranostics, Cascade dual activation, Lung cancer, Immunotherapy, Photodynamic therapy

## Abstract

Lung cancer is the most frequently diagnosed malignancy worldwide and remains the leading cause of cancer-related mortality. Effective treatment of immune-cold lung tumors remains particularly challenging due to poor immune activation and the off-target toxicity of conventional therapies. Here, we present a small-molecule–based self-assembled nanotheranostic micelle (FRANT) that employs cascade activation via folate receptor–mediated endocytosis and subsequent cathepsin B–specific cleavage, enabling tumor-selective near-infrared (NIR) fluorescence imaging and photodynamic therapy (PDT). FRANT maintains a serum-stable quenched state, thereby suppressing background fluorescence and minimizing off-target phototoxicity during systemic circulation. With an optimal hydrodynamic size of 13.7 nm, FRANT achieves deep tumor penetration, precise NIR fluorescence recovery, and robust singlet oxygen generation selectively in cancer cells. Importantly, FRANT-mediated PDT transformed immune-cold lung tumors by inducing immunogenic cell death and synergizing with PD-1 blockade, resulting in enhanced CD8⁺ T cell infiltration and durable tumor regression without systemic toxicity. Collectively, this study introduces a novel class of serum-stable, cascade-activated small-molecule nanotheranostics that couple diagnostic precision with immunomodulatory efficacy, offering a powerful platform for next-generation lung cancer therapy.

## Graphical abstract

**Figure:**
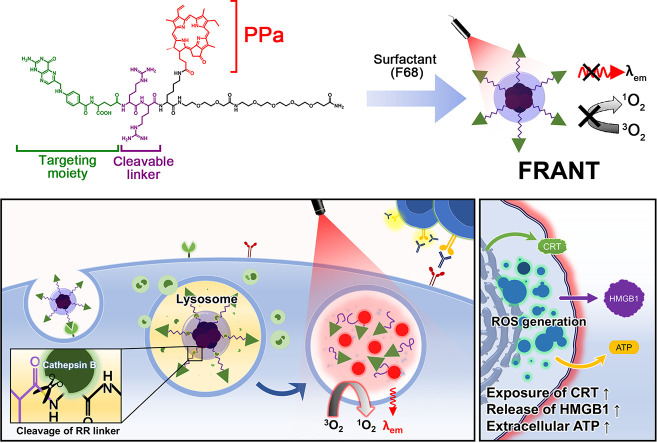

## 1 Introduction

According to the GLOBOCAN 2022 database, lung cancer is the most commonly diagnosed cancer and remains a leading cause of cancer-related mortality worldwide [1,2]. The International Agency for Research on Cancer (IARC) estimated approximately 2.5 million new cases and 1.8 million deaths in 2022 alone [2]. The poor prognosis of lung cancer is largely attributable to its rapid tumor growth and the absence of noticeable symptoms at early stages, leading to missed opportunities for timely detection. Currently, a wide range of treatment options, including surgery, chemotherapy and radiotherapy, are employed in the clinical management of lung cancer. However, these conventional treatments frequently cause severe adverse effects, which represent major obstacles to achieving curative outcomes [3].

More recently, immunotherapy using immune checkpoint inhibitors (ICIs), including antibodies targeting programmed death-ligand 1 (PD-L1) or programmed death 1 (PD-1), has emerged as a promising therapeutic modality, offering prolonged overall survival compared with platinum-based chemotherapy [4,5]. However, ICIs have shown limited efficacy in patients with large or “cold” tumors that lack pre-existing immune infiltration [6,7]. Additionally, their clinical application is restricted by factors such as the prohibitively high cost of treatment (exceeding USD 100,000 per patient annually) [8] and severe immune-related adverse events, including potentially life-threatening cardiotoxicities [9]. Therefore, the development of safe and cost-effective therapeutic strategies capable of eliciting potent antitumor responses with minimal systemic toxicity remains an urgent challenge in lung cancer therapy.

Photodynamic therapy (PDT) is an emerging therapeutic modality that induces rapid apoptosis and necrosis in cancer cells by producing abundant reactive oxygen species (ROS) upon irradiation with a specific wavelength of light [10]. Its therapeutic efficacy is achieved only in the simultaneous presence of a photosensitizer (PS), appropriate light and molecular oxygen, ensuring both safety and selectivity. Additionally, PDT facilitates the exposure of tumor-associated antigens and promotes immune cell infiltration into tumors, making it a promising strategy to trigger immunogenic cell death (ICD) and to potentiate the effects of immunotherapy [[11], [12], [13]]. Conventional PSs are classified as “always-on” types, exhibiting constant fluorescence or ROS generation regardless of their localization (i.e., in tumors or surrounding normal tissues). As a result, always-on PSs can cause severe off-target phototoxicity in adjacent normal tissues during light irradiation [14]. Furthermore, the limited tumor-targeting ability and unfavorable pharmacokinetics of conventional PSs often lead to prolonged skin photosensitivity, a well-recognized drawback of PDT [15]. To overcome these limitations, various activatable PSs have been developed, including pH-, enzyme- and redox-responsive systems [14,16]. Such activatable PSs remain inactive under normal physiological conditions but become highly phototoxic upon activation in the tumor microenvironment, thereby enhancing selectivity toward cancer cells while minimizing collateral damage.

Among the currently reported activatable PS designs, small-molecule activatable PSs with well-defined molecular structures hold significant promise for large-scale production and clinical translation. Yet, most second-generation PSs, particularly chlorin or phthalocyanine derivatives with high singlet oxygen (^1^O₂) quantum yields, are inherently hydrophobic, leading to poor dispersion stability in aqueous environments and difficulty in maintaining a quenched state under serum-rich conditions due to nonspecific interactions between hydrophobic PSs and serum proteins. In addition, activatable PSs that integrate cancer-targeting ligands within a small-molecule framework have been scarcely reported [[17], [18], [19]]. Thus, the development of small-molecule-based activatable PSs that incorporate targeting ligands, remain stably quenched in serum, and selectively activate fluorescence and phototoxicity in cancer cells remains a critical unmet need.

**Fig. 1:**
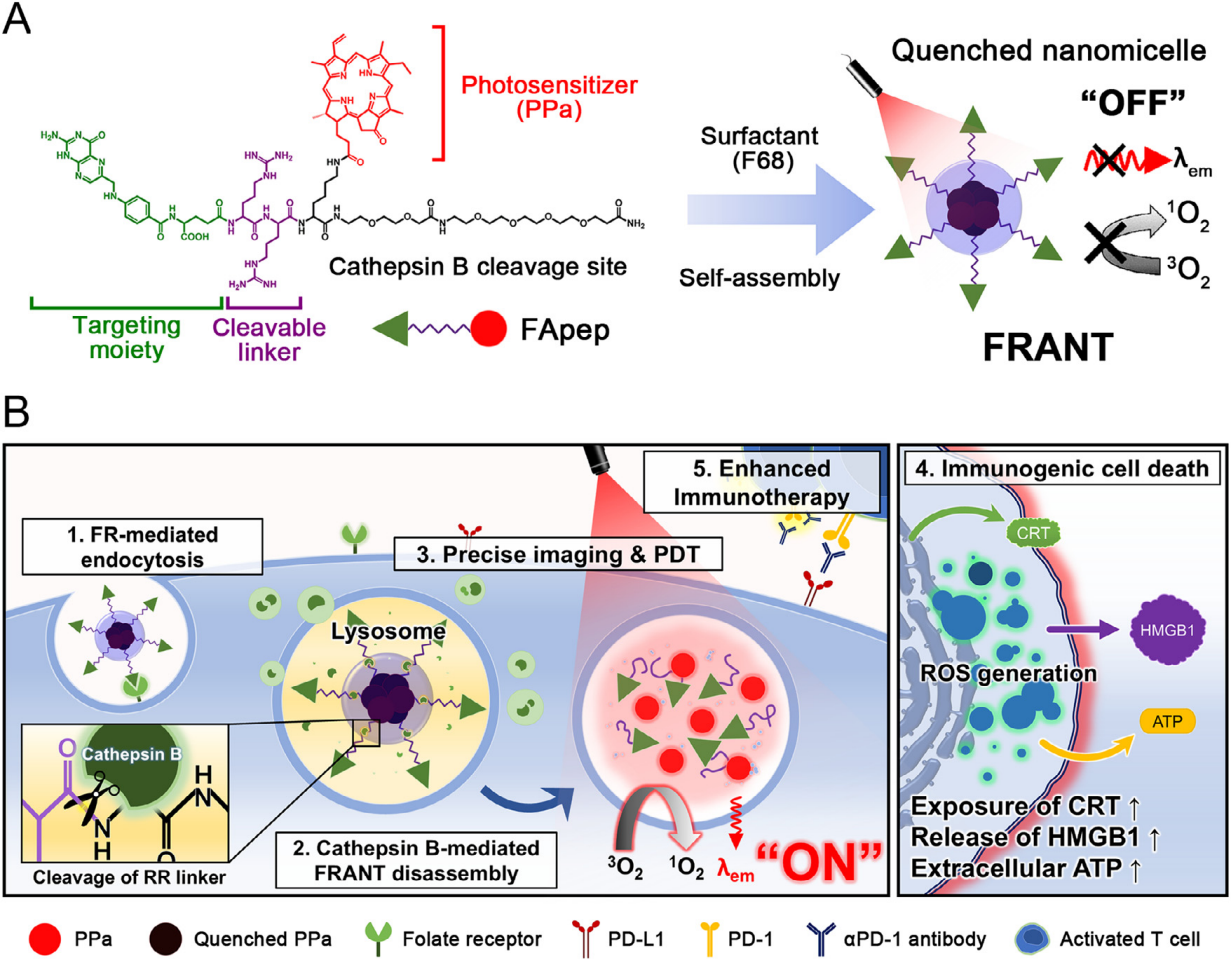
Schematic illustration of FRANT-based tumor imaging, PDT and synergistic immunotherapy. (A) FRANT is formed via self-assembly of FApep, an amphiphilic conjugate of folic acid and PPa linked through a cathepsin B-cleavable di-arginine peptide. In its assembled state, FRANT exhibits quenched NIR fluorescence and suppressed ^1^O_2_ generation (“OFF”); (B) Upon folate receptor (FR)–mediated endocytosis followed by cathepsin B–triggered cleavage, FRANT is activated, restoring NIR fluorescence and ^1^O₂ generation (“ON”). This activation enables tumor-selective imaging, PDT and ICD induction. When combined with immune checkpoint blockade, FRANT-mediated PDT synergistically enhances antitumor immunity, converting immune-cold lung tumors into immune-hot tumors.

In this work, we developed a cascade-activatable small-molecule nanotheranostic micelle (FRANT) that integrates dual-level selectivity for lung cancer therapy (Fig. 1A). The folate receptor is frequently overexpressed in lung cancer cells [20]. Additionally, cathepsin B is highly expressed in various human cancers, particularly in lung cancer, where its elevated activity contributes to tumor aggressiveness and correlates with increased lymph node metastasis and poor overall survival [21]. Therefore, the folate receptor and cathepsin B were employed as dual targets to achieve lung cancer–specific activation of the PS.

FRANT is constructed from an amphiphilic peptide conjugate (FApep), in which folic acid (FA), a targeting ligand for folate receptor-overexpressing cancer cells, is covalently linked to the PS pyropheophorbide-a (PPa) via a di-arginine peptide linker that is specifically cleavable by cathepsin B. Short PEGylation enhances aqueous solubility and promotes self-assembly with Pluronic F68, yielding nanomicelles with a uniform hydrodynamic size of ∼13.7 nm. It has been reported that a size range of 7–20 nm enables improved tumor penetration, high cellular internalization and prolonged blood circulation, suggesting that FRANT, operating within this optimal size range, holds promise as an effective tumor treatment modality [[22], [23], [24], [25]]. Importantly, FRANT maintains a serum-stable quenched state, thereby suppressing background fluorescence and minimizing off-target phototoxicity during systemic circulation. Following folate receptor–mediated endocytosis, the di-arginine linker is cleaved by lysosomal cathepsin B, triggering nanomicelle disassembly and restoring both near-infrared (NIR) fluorescence and ^1^O₂ generation selectively within tumor cells (Fig. 1B).

This cascade activation strategy not only enables tumor-specific imaging and precisely controlled photodynamic ablation but also induces tumor-selective ICD, thereby facilitating immune cell infiltration (Fig. 1B). When combined with PD-1 blockade, FRANT-mediated PDT effectively converts immune-cold lung tumors into immunologically “hot” tumors, eliciting robust antitumor immunity and durable tumor regression without systemic toxicity. Thus, FRANT represents a novel class of small-molecule nanotheranostics that unifies diagnostic precision, cascade-controlled activation and immunomodulatory efficacy, providing a promising platform for next-generation lung cancer treatment.

## 2 Materials and methods

### 2.1 Materials

Dimethyl sulfoxide (DMSO), Pluronic F68 (F68), folic acid (FA), D,L-dilhiothreitol (DTT), chloroform, trifluoroacetic acid (TFA) and acetonitrile (ACN) were purchased from Merck KGaA (Darmstadt, Germany). Dialysis tubes (molecular weight cut-off: 3.5 kDa) and Amicon centrifugal filters (molecular weight cut-off: 50 kDa) were also obtained from Merck KGaA. PPa was purchased from Frontier Scientific (Logan, UT, USA). Phosphate-buffered saline (PBS) and Dulbecco's Modified Eagle's Medium (DMEM) without phenol red were obtained from Gibco (NY, USA). Primary antibodies against cluster of differentiation 8 (CD8), high-mobility group box 1 (HMGB1), an Alexa Fluor 488–conjugated anti-rabbit secondary antibody, as well as 2′,7′-dichlorofluorescin diacetate (DCF-DA) were purchased from Abcam (Cambridge, UK). Alexa Fluor 488–conjugated anti-calreticulin antibody was purchased from Cell Signaling Technology (MA, USA). RealTime-Glo extracellular adenosine triphosphate (ATP) assay kit was purchased from Promega (MD, USA).

### 2.2 Preparation of FRANT

FApep was synthesized by Hefei KS-V Peptide Biological Technology Co., Ltd. (Hefei, China). The purity and mass of the synthesized FApep were analyzed by the company. For the preparation of FRANT, FApep in DMSO (0.5 ml, 20 mg/ml), and F68 in deionized water (DW, 0.5 ml, 10 mg/ml) were mixed and vortexed to achieve a molar ratio of FApep to F68 of 1:0.2. The mixture was then transferred to a dialysis tube (molecular weight cut-off: 3.5 kDa) and dialyzed against DW for 24 h to form self-assembled nanomicelles. After dialysis, the solution was placed in an Amicon centrifugal filter (molecular weight cut-off: 50 kDa) and centrifuged at 3800 rpm for 10 min to concentrate the final product and remove residual impurities. Finally, the obtained FRANT was stored at 4 °C until use.

### 2.3 Characterization of FApep and FRANT

To compare the characteristic peaks of the ultraviolet–visible (UV-Vis) spectra of free PPa, FApep and FA, all samples were dissolved in DMSO at a 5 µM equivalent PPa concentration (for free PPa and FApep) and a 20 µM concentration (for FA). UV-Vis spectra were then obtained using a UV-Vis spectrophotometer (UV-1200, Labentech, Incheon, Korea). To identify differences in the UV-Vis spectra between FApep and FRANT, PPa was dissolved in DMSO, and FRANT was dispersed in DW at a 10 µM equivalent PPa concentration. The UV-Vis spectra of both samples were measured using a UV-Vis spectrophotometer.

The hydrodynamic size of FRANT was measured using a Zetasizer Nano ZS (Malvern Instruments Ltd., Malvern, UK). FRANT was diluted in saline to a 10 µM equivalent PPa concentration. The time-dependent dispersion stability of FRANT was also evaluated by measuring its hydrodynamic size at 0 d, 7 d, 12 d, 19 d and 33 d The particle size of dried FRANT was investigated using transmission electron microscopy (TEM). A TEM image of FRANT was obtained at an acceleration voltage of 120 kV using a Talos L120C (FEI, Hillsboro, OR, USA).

The quenching of PPa fluorescence in FRANT was investigated by measuring fluorescence spectra and NIR fluorescence imaging of free PPa and FRANT. To prepare the non-quenched control, free PPa was dissolved in DMSO. To evaluate the fluorescence quenching of FRANT under physiological conditions, FRANT was dispersed in PBS and in DMEM without phenol red media containing 10% fetal bovine serum (FBS, Gibco, NY, USA). All samples were prepared at a 5 µM PPa-equivalent concentration. The fluorescence spectra of the samples were then compared using a multifunctional microplate reader (SPARK; TECAN Trading AG, Zurich, Switzerland) with an excitation wavelength of 360/35 nm. Additionally, NIR fluorescence images of free PPa and FRANT were obtained with an excitation wavelength of 620/20 nm and an emission wavelength of 670/40 nm using an IVIS Lumina XRMS (Xenogen Corporation-Caliper, CA, USA).

The quenching stability of FRANT was evaluated in PBS and 10% FBS-containing DMEM. FRANT was dispersed in PBS and 10% FBS-containing DMEM at a 5 µM PPa-equivalent concentration. A free PPa solution in DMSO was also prepared at the same concentration as FRANT, serving as a control. The samples were then placed on a 96-well plate, and their fluorescence intensities (λ_ex._ = 360/5 nm, λ_em._ = 665/8 nm) were measured every hour for 16 h using a multifunctional microplate reader.

The singlet oxygen generation (SOG) of free PPa and FRANT was evaluated using the Singlet Oxygen Sensor Green (SOSG, Thermo Fisher Scientific, CA, USA) reagent. Free PPa in O₂-saturated 1% SDS-containing PBS was mixed with 0.5 µL of 1 mM SOSG reagent to achieve a 5 µM PPa concentration. FRANT in O₂-saturated PBS was mixed with 0.5 µl of 1 mM SOSG reagent to achieve a 5 µM PPa-equivalent concentration. A control sample containing only SOSG reagent was prepared by mixing 0.5 µl of 1 mM SOSG reagent with 499.5 µl O₂-saturated PBS. The samples were then irradiated every 10 s for 60 s using a 670 nm continuous wave (CW) laser at a power density of 50 mW/cm^2^. The fluorescence intensities (λ_ex._ = 485/20 nm, λ_em._ = 540/25 nm) of the irradiated samples were measured at each time point using a multifunctional microplate reader.

### 2.4 Characterization of enzymatic cleavage of FRANT

The cleavage of the di-arginine linker in FRANT by cathepsin B was analyzed by high-performance liquid chromatography (HPLC, Alliance, Waters Corporation, MA, USA). FApep, its synthesized cleaved product (K(PPa)-miniPEG), and cathepsin B-treated FRANT were analyzed. FApep and K(PPa)-miniPEG were dissolved in DMSO at a 2 µM equivalent PPa concentration. For the preparation of cathepsin B-treated FRANT, FRANT was mixed with 50 mM sodium acetate buffer (pH 5.2) containing 1 unit of cathepsin B and 5 mM DTT to achieve a final equivalent PPa concentration of 2 µM. The fluorescence intensity of cathepsin B-treated FRANT reached its maximum at 4 h (data not shown) due to dequenching of PPa fluorescence following cleavage of the di-arginine linker in FRANT. Therefore, the mixture was then agitated using a thermomixer at 300 rpm and 37 °C for 4 h for the enzymatic reaction. After the reaction, the enzyme-treated FRANT solution was mixed with an equal volume of chloroform and vortexed for 1 min. The mixture was then allowed to stand at 25 °C for 30 min to facilitate phase separation. The chloroform layer was collected and the organic solvent was evaporated. The residue was re-dissolved in DMSO. The chromatograms of the samples were analyzed using HPLC with a mobile phase consisting of 0.08% TFA in DW (eluent A) and 0.08% TFA in ACN (eluent B) at a flow rate of 1.0 ml/min. The elution gradient was set as follows: 80% A/20% B at 0 min, 80% A/20% B at 2 min, 5% A/95% B at 40 min, and 5% A/95% B at 50 min. The sample was separated using an XBridge Peptide BEH C_18_ column (300 Å, 5 µm, 4.6 mm × 250 mm, Waters Corporation, MA, USA) and detected using a fluorescence detector (λ_ex_ = 400 nm, λ_em_ = 663 nm).

### 2.5 Cell culture

The Lewis lung carcinoma-1 (LLC-1) murine lung cancer cell line (RRID: CVCL_4358) was obtained from the American Type Culture Collection (ATCC, Rockville, MD, USA) in 2024. The cells were cultured in DMEM (Gibco) supplemented with 10% FBS (Gibco) and 1% penicillin-streptomycin (Gibco, NY, USA).

### 2.6 In vitro cellular evaluation of cascade-activatable NIR fluorescence imaging of FRANT in lung cancer cells

LLC-1 cells were seeded in 8-well Lab-Tek II chambered coverglasses at a density of 5 × 10^4^ cells per well. To evaluate the fluorescence activation of FRANT within cancer cells, LLC-1 cells were incubated with either free PPa or FRANT at a concentration equivalent to 5 µM PPa for 2 h. The cells were then washed with Dulbecco’s phosphate-buffered saline (DPBS, Gibco, NY, USA) to remove extracellular residues. Confocal fluorescence images (λ_ex._ = 633 nm, λ_em._ = 663 nm) were subsequently acquired at 0, 2, 4 and 6 h post-washing using a confocal laser scanning microscope (LSM 780, ZEISS, Oberkochen, Germany).

To analyze folate receptor–mediated endocytosis and subsequent cathepsin B–mediated fluorescence activation of FRANT inside cancer cells, a separate set of LLC-1 cells was prepared under the same seeding conditions. These cells were incubated with either free PPa (5 µM) or FRANT (5 µM PPa-equivalent, corresponding to 5 µM FA) for 4 h. After washing with DPBS, the cells were further incubated in fresh culture medium for 2 h. For the inhibition of receptor–mediated endocytosis and enzyme–mediated fluorescence activation, LLC-1 cells were pretreated with either 1 mM excess FA or 100 µM of the membrane-permeable cathepsin B inhibitor E64d (Merck KGaA, Darmstadt, Germany) for 30 min prior to incubation with free PPa or FRANT. Additionally, treated cells were co-stained with LysoTracker (Invitrogen, CA, USA) for 1 h. After two washes with DPBS, fluorescence images (PPa: λ_ex._ = 633 nm, λ_em._ = 663 nm; LysoTracker, λ_ex._ = 373 nm, λ_em._ = 422 nm) were acquired using a confocal laser scanning microscope.

### 2.7 In vitro dark toxicity and phototoxicity test

LLC-1 cells were seeded in 96-well plates at a density of 5 × 10^3^ cells per well and incubated overnight at 37 °C in a 5% CO_2_ incubator. The cells were treated with either free PPa or FRANT at varying concentrations (0, 0.625, 1.25, 2.5, 5 and 10 µM PPa-equivalent) for 4 h. Following drug treatment, the cells were washed twice with DPBS and incubated with fresh culture medium for an additional 2 h. For dark toxicity assessment, cells were not exposed to light, and cell viability was measured 24 h later using the CellTiter-Glo (CTG) Luminescent Cell Viability Assay (Promega, MD, USA). For phototoxicity evaluation, cells were irradiated with a 670 nm CW laser (50 mW/cm^2^, 200 s), followed by a 24 h incubation. Cell viability was then measured using the CTG assay.

To investigate folate receptor–mediated endocytosis and cathepsin B–mediated photodynamic activation of FRANT, inhibition tests were conducted. LLC-1 cells were pretreated with 100 µM E64d or 1 mM excess FA for 30 min. Subsequently, the cells were incubated with a 5 µM PPa-equivalent concentration of free PPa or FRANT for 4 h, washed with DPBS, and incubated with fresh medium for 2 h. Cells without FA or E64d pretreatment were used as controls. All groups were then irradiated with the CW laser (50 mW/cm^2^, 200 s). After a further 24 h incubation, cell viability was assessed using the CTG assay. Untreated cells (no drug, no irradiation) served as the control group and were defined as 100% viable.

### 2.8 In vitro cellular evaluation of cascade-activatable ROS generation in FRANT-treated lung cancer cells

Cascade-activatable intracellular ROS generation in FRANT-treated lung cancer cells was evaluated following PDT. LLC-1 cells were seeded in a 24-well black-walled plate (SPL life Sciences, Gyeonggi-do, Korea) at a density of 3 × 10^4^ cells per well and incubated for 2 d at 37 °C in a 5% CO_2_ incubator. The cells were incubated with FRANT at a concentration equivalent to 5 µM PPa for 4 h in the presence or absence of 100 µM E64d or 1 mM excess FA. They were then washed with DPBS and incubated in fresh medium for an additional 2 h. To visualize intracellular ROS generation, the FRANT-treated cells were stained with 40 µM DCF-DA in phenol red-free DMEM. Subsequently, all treatment groups were irradiated with a 670 nm CW laser at a power density of 50 mW/cm^2^ for a total dose of 10 J/cm^2^. After a 45 min incubation, the cells were washed three times with DPBS to remove residual extracellular dye. Fluorescence images of intracellular DCF (λ_ex._ = 485 nm, λ_em._ = 535 nm) were acquired using an LSM 780 confocal microscope.

### 2.9 In vitro cellular evaluation of cascade-activatable induction of ICD in FRANT-treated lung cancer cells

The ICD-related markers, including extracellular ATP (eATP), calreticulin exposure and extranuclear and extracellular HMGB1 release, were evaluated in PDT-treated LLC-1 cells with or without pretreatment with 100 µM E64d or 1 mM excess FA. The level of eATP was measured using a commercial luminescence-based assay kit (Promega) in accordance with the manufacturer’s instructions. Temporal changes in eATP release were measured, and differences among treatment groups at the peak level were quantitatively analyzed.

To assess cell-surface exposure of calreticulin, PDT-treated LLC-1 cells were detached using Accutase solution (Sigma-Aldrich, St. Louis, MO, USA) and resuspended in cold FACS buffer (Invitrogen, *CA*, USA). The cells were stained with an Alexa Fluor 488–conjugated anti-calreticulin antibody (1:200, Cell Signaling Technology) for at least 30 min at 25 °C in the dark. The stained cells were analyzed by flow cytometry using a FACSLyric II flow cytometer (BD Biosciences, NJ, USA). Data were processed using FlowJo software (BD Biosciences, NJ, USA).

HMGB1 release was visualized by immunofluorescence staining. PDT-treated LLC-1 cells were permeabilized with 80% methanol and incubated with an anti-HMGB1 primary antibody (1:200, Abcam), followed by an Alexa Fluor 488–conjugated secondary antibody (1:400, Abcam). Fluorescence images were acquired using an LSM 780 confocal microscope.

### 2.10 LLC-1 syngeneic tumor mouse model

Female C57BL/6 mice were obtained from Orient Bio Inc. (Gyeonggi-do, Korea). All animal procedures were conducted in accordance with the guidelines of the Institutional Animal Care and Use Committee of the National Cancer Center Research Institute (IACUC Approval No. NCC-23-916-001). LLC-1 cells (5 × 10^5^ cells in 50 µL Hanks' Balanced Salt Solution) were subcutaneously injected into the dorsal flank of each mouse. Tumor volumes were measured every 2 d using a digital caliper.

### 2.11 In vivo NIR fluorescence imaging

Free PPa was first dissolved in DMSO (2 mg/mL), then diluted in a solution containing 2% (v/v) ethanol, 1% (v/v) Tween 80, 0.2 mM sodium bicarbonate, and 5% (w/v) dextrose to prepare a water-soluble PPa formulation, as previously described [26]. FRANT was dispersed in saline at a concentration of 5 mg PPa-equivalent per kg body weight in 50 µl. When the tumor volume in C57BL/6 mice reached ∼100 mm^3^, mice were intravenously injected with either free PPa or FRANT at a dose of 5 mg PPa-equivalent per kg body weight (*n* = 3 per group). A control group (*n* = 3) received an intravenous injection of saline. NIR fluorescence images (λ_ex._ = 620/20 nm, λ_em._ = 670/40 nm) were acquired using the IVIS Lumina XRMS system (Xenogen Corporation-Caliper, CA, USA) at 20 min, 2 h and 24 h post-injection. At 24 h post-injection, all mice were sacrificed, and major organs (heart, spleen, lung, liver, and kidney) along with tumor tissues were harvested. *Ex vivo* fluorescence imaging of the collected tissues was performed and analyzed.

### 2.12 Antitumor effect of FRANT in combined PDT and immunotherapy

To evaluate the antitumor effect in a mouse model, free PPa and FRANT samples were prepared in the same way as the *in vivo* imaging study. When tumor volumes reached ∼100 mm³, the mice were randomly divided into ten groups (*n* = 7 per group) as follows: Group 1, PBS control (100 µL); Group 2, free PPa (5 mg/kg); Group 3, free PPa (5 mg/kg) + light irradiation (L); Group 4, FRANT (2 mg/kg); Group 5, FRANT (2 mg/kg) + L; Group 6, FRANT (5 mg/kg); Group 7, FRANT (5 mg/kg) + L; Group 8, αPD-1 monotherapy (αPD-1, 10 mg/kg); Group 9, αPD-1 (10 mg/kg) + FRANT (2 mg/kg) + L; Group 10, αPD-1 (10 mg/kg) + FRANT (5 mg/kg) + L. On Day 1, tumor-bearing mice received an intravenous injection of FRANT (2 or 5 mg/kg) or free PPa (5 mg/kg). PDT was performed on Day 2 by irradiating the tumor sites with a 670 nm CW laser at 50 mW/cm^2^ for a total dose of 20 J/cm^2^ (Fig. 7A). For immunotherapy, αPD-1 (10 mg/kg was administered intraperitoneally every 2 d from Day 1 to 9. The antitumor study was terminated on Day 11, when the tumor volume in the control group exceeded 1000 mm^3^. The *in vivo* synergistic effect of the combination therapy was analyzed by calculating the coefficient of drug interaction (CDI) as previously reported [27]. CDI values of <1, <0.7, =1 and >1 indicate synergy, significant synergy, additivity and antagonism, respectively. All mice were euthanized in accordance with institutional animal care and ethical guidelines.

### 2.13 Histological analysis of tumor tissues

To analyze tumor apoptosis, immune cell infiltration and ICD induction following PDT and immunotherapy, an additional cohort of tumor-bearing mice was prepared. When tumor volumes reached ∼100 mm^3^, the mice were randomly divided into nine groups (*n* = 3 per group) and received the same treatments as described above. As the tumor size in Group 10 was too small for tissue sectioning on Day 3, this group was excluded from the analysis. On Day 3 (i.e., 24 h after PDT), tumor tissues from all groups were harvested and fixed in 4% paraformaldehyde (T&I Biotechnology, Chuncheon, Korea), followed by paraffin embedding and tissue sectioning.

Tissue sections for apoptosis analysis were stained using a terminal deoxynucleotidyl transferase-mediated dUTP nick-end labeling (TUNEL) assay kit. For immune cell infiltration and ICD analysis, tissue sections were subjected to heat-mediated antigen retrieval using Tris/EDTA buffer (pH 9.0), followed by immunostaining with primary antibodies against CD8 (1:2000), HMGB1 (1:1000) and calreticulin (1:3000). Quantitative analysis of apoptosis and CD8^+^ T cell infiltration was performed using seven stained images for apoptotic cells and eight stained images for CD8^+^ T cells. The number of apoptotic cells and CD8^+^ T cells per image was counted using ImageJ software (National Institutes of Health, MD, USA) to assess differences between treatment groups.

### 2.14 Safety of FRANT and treatment regimen in vivo

To evaluate the potential toxicity of FRANT and the treatment regimen, major organs (i.e., heart, lung, kidney, spleen, and liver) were collected from the mice on Day 11, sectioned and stained with hematoxylin and eosin (H&E) to assess histopathological changes. Blood samples were also collected, and serum biochemical analyses were performed.

### 2.15 Statistical analysis

Student’s *t*-test was used to analyze significant differences between groups. All data are expressed as the mean ± standard error (S.E.).

## 3 Results and discussion

### 3.1 Preparation and characterization of FRANT

**Fig. 2:**
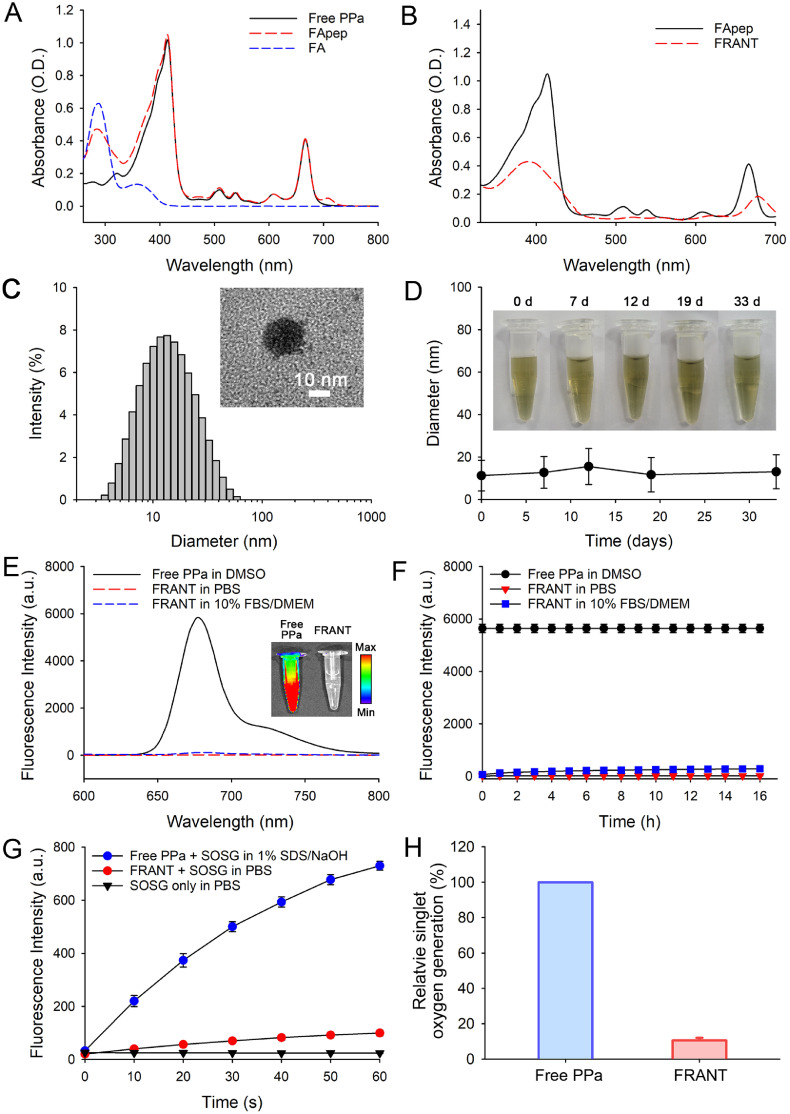
Characterization of FRANT. (A) UV-Vis absorption spectra of free PPa and FApep in DMSO at 5 μM. For comparison, the UV-Vis absorption spectrum of FA at 20 μM is also shown; (B) UV-Vis absorption spectra of FApep in DMSO and FRANT in water at a PPa-equivalent concentration of 10 μM; (C) Hydrodynamic size and TEM image (inset) of FRANT; (D) Hydrodynamic size stability of FRANT over 33 d (*n* = 3); (E) Fluorescence spectra (λ_ex._ = 360/35 nm) of FRANT in PBS and 10% FBS-containing DMEM at a PPa-equivalent concentration of 5 μM. The fluorescence spectrum of free PPa in DMSO at 5 μM is shown for comparison. The inset displays NIR fluorescence images (λ_ex._ = 620/20 nm, λ_em._ = 670/40 nm) of microtubes containing PPa in DMSO and FRANT in PBS at a 5 μM PPa-equivalent concentration; (F) NIR fluorescence quenching stability of FRANT in PBS and 10% FBS-containing DMEM. Free PPa in DMSO was used as a control (*n* = 3 per group); (G) Time-dependent ^1^O₂ generation comparison under 670 nm CW laser irradiation (50 mW/cm^2^) for free PPa, FRANT, and SOSG only, at a 5 μM PPa-equivalent concentration (*n* = 3 per group); (H) Relative ¹O₂ generation of free PPa and FRANT, calculated at the 60 s time point (*n* = 3 per group).

UV-Vis absorption spectra of samples obtained in DMSO owed to their poor water solubility. The UV-Vis absorption spectrum of free PPa exhibited the characteristic Soret and Q bands of PPa at 414 and 667 nm, respectively (Fig. 2A) [28]. FA displayed a strong absorption band at 286 nm, attributed to the π–π* transition of the pterin ring [29]. FApep showed strong absorption bands at 286, 414 and 667 nm, corresponding to the π–π* transition of FA and the Soret and Q bands of PPa, respectively. These spectral features confirm the successful conjugation between PPa and FA.

The purity and molecular weight of FApep were further verified using HPLC–mass spectrometry (MS). The HPLC chromatogram of FApep showed a single peak with an elution time of 14.5 min and a purity of 95.2% (Fig. S1A). Subsequent MS analysis of the eluted FApep revealed a molecular weight of 1790.9 g/mol, consistent with the theoretical molecular weight (Fig. S1B).

FApep was designed as an amphiphilic peptide to form nanomicelles via self-assembly in aqueous solution in the presence of F68 surfactant. FApep contains both hydrophobic moieties (PPa and FA) and hydrophilic components (di-arginine peptide substrate and miniPEG tail), enabling self-assembly in aqueous environments. In preliminary studies, FApep alone exhibited a critical micelle concentration of approximately 970 ng/ml. However, when dissolved at a high concentration (10 mg/ml) and dialyzed into aqueous solution—conditions required to prepare sufficiently concentrated formulations for *in vivo* studies—FApep alone formed large and heterogeneous aggregates (data not shown). To obtain smaller and more uniform nanomicelles suitable for systemic administration, F68 was incorporated as a stabilizing amphiphilic polymer. In this architecture, FApep primarily drives hydrophobic core formation through π–π stacking and hydrophobic interactions, whereas F68 functions as a steric stabilizer that improves size control and colloidal stability.

The concentration of F68 in FRANT was determined using the cobalt thiocyanate method with slight modification (in Supplementary Materials). Quantitative analysis revealed that the prepared FRANT consisted of FApep and F68 with a molar ratio of 1:0.07 (Table S1). The UV-Vis absorption spectrum of FRANT in water was compared with that of FApep in DMSO (Fig. 2B). The Soret and Q bands of FRANT were noticeably broadened relative to those of FApep in DMSO. This spectral broadening suggests aggregation of hydrophobic PPa molecules within FRANT [28,30].

The particle size of FRANT was characterized by dynamic light scattering (DLS) and TEM. The average hydrodynamic diameter of FRANT was 13.7 ± 7.4 nm, consistent with the particle size observed in the TEM image (Fig. 2C). To evaluate colloidal stability, the hydrodynamic size of FRANT was monitored for 33 d and remained stable at ∼14 nm without detectable aggregation. Consistent with these measurements, visual observation confirmed that the dispersion remained clear, without agglomeration or sedimentation, throughout the 33 d period (Fig. 2D).

The size of nanomicelles is a critical factor for effective cancer therapy. Nanoparticles within the 7–20 nm size range benefit from enhanced tissue penetration owing to reduced diffusion resistance, and nanoparticles smaller than 20 nm can overcome osmotic barriers imposed by interstitial fluid pressure and the extracellular matrix [22]. Nanoparticles with sizes ranging from 12 to 50 nm have been reported to be effective for cellular uptake and optimal transport, supporting their clinical applicability for solid tumor treatment [23,25,31]. Nanoparticles smaller than approximately 9 nm are susceptible to rapid renal clearance and urinary excretion, whereas those larger than 10 nm exhibit prolonged blood circulation, resulting in enhanced tumor accumulation [32,33]. Moreover, compared with nanoparticles larger than 100 nm, which are preferentially sequestered by reticuloendothelial system (RES) organs such as the liver, nanoparticles within this smaller size range demonstrate reduced hepatic accumulation [25]. Collectively, these considerations suggest that FRANT nanomicelles with particle sizes in the 10–20 nm range are favorable for effective tumor therapy. The particle size of FRANT was determined to be 13.7 nm by DLS and TEM, conferring an advantage in reaching the central regions of tumors through efficient tissue penetration.

The fluorescence properties of FRANT in PBS and in DMEM containing 10% FBS were then analyzed. For comparison, the fluorescence spectrum of free PPa in DMSO was also recorded (Fig. 2E). Free PPa in DMSO exhibited a strong emission band at 678 nm. By contrast, FRANT exhibited markedly quenched fluorescence in PBS and remained effectively quenched when dispersed in DMEM containing 10% FBS. NIR fluorescence imaging of microtubes containing free PPa and FRANT showed a similar trend, corroborating the spectral data. The pronounced quenching of NIR fluorescence in FRANT was ascribed to the aggregation of PPa molecules within the nanomicelle core, and this quenched state was maintained even in the presence of serum proteins.

The maintenance of fluorescence quenching in FRANT is critical to ensure that the activatable PS is selectively activated in target tissues. To evaluate this stability, changes in fluorescence intensity were monitored over 16 h at 37 °C. The fluorescence intensities of FRANT in both PBS and 10% FBS-containing DMEM remained considerably lower than those of free PPa in DMSO throughout the 16 h incubation (Fig. 2F). These results indicate that the quenched state of FRANT was stably maintained even in the presence of serum proteins.

As mentioned above, most PSs used in activatable PDT designs are inherently hydrophobic, which leads to poor dispersion stability in aqueous environments and difficulty in maintaining a quenched state under serum-rich conditions owing to nonspecific interactions with serum proteins. Notably, fluorescence quenching in FRANT persisted for over 16 h at 37 °C, even in the presence of abundant serum proteins (Fig. 2F).

Generally, PSs undergo excitation from the S₀ state to the S₀ state via one-photon transition, followed by relaxation to the ground state through fluorescence emission. This process involves intersystem crossing, which leads to ROS production through Type I and Type II reactions [34]. However, aggregation-induced quenching of PPa fluorescence within FRANT prevents excitation of the PS from the S_0_ to the S_n_ state via one-photon transition, thereby inhibiting intersystem crossing and ROS generation. Therefore, the prolonged quenching of NIR fluorescence in FRANT observed in PBS and DMEM containing 10% FBS indicates that its quenched state, and consequently the suppression of ROS generation, can be maintained for at least 16 h at 37 °C, even under serum-rich conditions such as those of the bloodstream.

Since aggregation-induced fluorescence quenching can directly affect the efficiency of ^1^O₂ generation by PSs, the ^1^O_2_ generation of FRANT under 670 nm CW laser irradiation was evaluated and compared with that of free PPa (Figs. 2G and H). Notably, FRANT exhibited an approximately nine-fold lower ^1^O_2_ yield than free PPa, demonstrating effectively suppressed phototoxicity in its quenched state.

### 3.2 In vitro cellular evaluation of cascade-activatable NIR fluorescence imaging and PDT of FRANT in lung cancer cells

In activatable PDT design, recovery of quenched NIR fluorescence and ROS generation by PS in response to tumor-associated stimuli is crucial for achieving antitumor activity. To this end, the peptide substrate within FRANT must be cleaved by cathepsin B to induce disaggregation of PPa molecules, followed by dequenching of NIR fluorescence and restoration of phototoxicity. In fact, this “turn-on” strategy of FRANT activation by lysosomal cathepsin B carries significant implications for cancer therapy research and clinical applications. The high expression of cathepsin B in human cancers, particularly in lung cancer cells, has been reported to contribute to tumor aggressiveness, making it a potent prognostic factor [21]. Hence, we incorporated a cathepsin B-cleavable di-arginine linker into the FApep structure to enable lung cancer–specific activation of the PS.

**Fig. 3:**
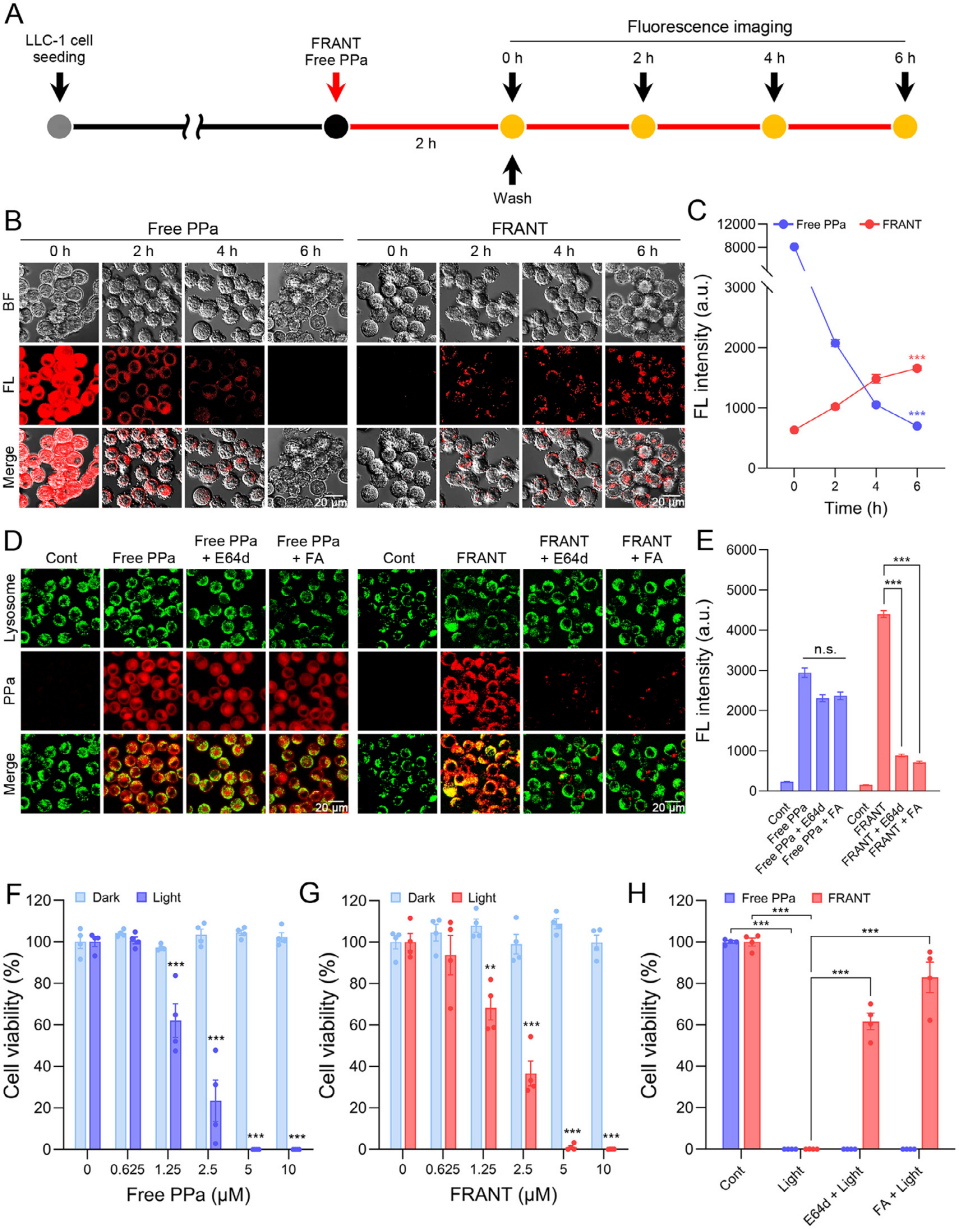
*In vitro* cellular evaluation of FRANT for cascade-activatable NIR fluorescence imaging and PDT in lung cancer cells. (A) Experimental scheme for evaluating intracellular fluorescence recovery of quenched FRANT in LLC-1 cells; (B) Fluorescence images of free PPa- and FRANT-treated LLC-1 cells at different time points after washing. The red channel indicates PPa fluorescence. Scale bars: 20 μm; (C) Quantitative analysis of intracellular PPa fluorescence from (B). Mean fluorescence intensity per cell was analyzed in >90 cells per group; (D) Fluorescence images of free PPa- and FRANT-treated LLC-1 cells in the absence or presence of the cathepsin B inhibitor E64d or excess FA. The red and green channels indicate fluorescence signals from PPa and LysoTracker, respectively. Scale bars*:* 20 μm; (E) Quantitative analysis of PPa fluorescence intensity from the images in (D). Mean fluorescence intensity per cell was analyzed in >170 cells per group; (F, G) Cell viability of LLC-1 cells treated with (F) free PPa or (G) FRANT at various concentrations under light irradiation, evaluating concentration-dependent phototoxicity (*n* = 4 per group); (H) Cell viability of LLC-1 cells treated with free PPa or FRANT at 5 μM PPa-equivalent under PDT conditions in the presence or absence of E64d or excess FA (*n* = 4 per group). Data are expressed as mean ± S.E. Statistical significance was determined using Student’s *t*-test (^⁎⁎^*P* < 0.01, ^⁎⁎⁎^*P* < 0.001).

Cancer-selective cascade activation of NIR fluorescence and phototoxicity of FRANT was assessed in the non-small cell lung cancer cell line LLC-1, which overexpresses folate receptors and lysosomal cathepsin B (Fig. S2A and S2B) [20,35]. LLC-1 cells were incubated with either free PPa or FRANT for 2 h. After extracellular compounds were removed by washing, intracellular fluorescence was monitored over time to evaluate the recovery of quenched NIR fluorescence within cancer cells (Fig. 3A). Free PPa-treated cells exhibited strong fluorescence at early time points, indicating efficient cellular uptake (Fig. 3B). However, the fluorescence signal progressively declined and was markedly reduced by 6 h post-washing, suggesting efflux of PPa from the cells. By contrast, FRANT-treated cells initially showed minimal fluorescence immediately after washing, indicating that intracellular FRANT maintained its quenched state at this time point. However, intracellular fluorescence of FRANT-treated cells progressively increased and reached a maximum at 6 h post-washing (Fig. 3B and 3C). These results demonstrate that the quenched NIR fluorescence of FRANT was restored inside cells via enzymatic cleavage of FApep, followed by release and dequenching of the conjugated PSs.

To further investigate the roles of cathepsin B and folate receptors in fluorescence recovery, LLC-1 cells were treated with free PPa or FRANT in the presence of E64d (a cathepsin B inhibitor) or excess FA (a competitive inhibitor of folate receptor binding). As shown in Fig. 3D and 3E, no appreciable change in fluorescence intensity was observed in free PPa-treated cells upon co-treatment with either E64d or excess FA. This result indicates that the fluorescence of free PPa is independent of cathepsin B– or folate receptor–mediated interactions. By contrast, intracellular fluorescence in FRANT-treated cells was markedly reduced upon E64d treatment (5.1-fold decrease, *P* < 0.001), confirming that the strong fluorescence observed in FRANT-treated cells was attributable to cathepsin B–mediated cleavage of the di-arginine linker and subsequent release of the PSs. Furthermore, co-treatment of FRANT (5 µM PPa-equivalent) with excess FA (1 mM) to inhibit folate receptor binding resulted in markedly reduced intracellular fluorescence compared with FRANT alone. Co-staining with LysoTracker further demonstrated localization of FRANT in lysosomal compartments, where cathepsin B is abundant. Together, these results demonstrate that FRANT is internalized into LLC-1 cells via folate receptor–mediated endocytosis, followed by fluorescence recovery through the enzymatic action of cancer-associated cathepsin B.

The roles of cathepsin B and the folate receptor in the cancer-selective PDT effect were then investigated. When LLC-1 cells were incubated with either free PPa or FRANT without light irradiation, no detectable cytotoxicity was observed at concentrations up to 10 µM PPa-equivalent (Fig. 3F and 3G). Upon irradiation with a 670 nm CW laser (50 mW/cm^2^, 200 s), however, both free PPa- and FRANT-treated cells exhibited dose-dependent phototoxicity. The IC_50_ values were 1.10 µM for free PPa and 1.89 µM for FRANT. Considering that the SOG of FRANT is considerably lower than that of free PPa (Fig. 3E), the comparable PDT efficacy of FRANT suggests that SOG is restored within cancer cells.

We therefore investigated whether the high PDT efficacy of FRANT was attributable to receptor–mediated cellular uptake and subsequent recovery of SOG by intracellular cathepsin B. To this end, LLC-1 cells were pretreated with either E64d or excess FA, followed by treatment with free PPa or FRANT and subsequent irradiation with a 670 nm CW laser. Notably, cell viability increased to 61.7% in E64d-pretreated cells and to 82.9% in excess FA-pretreated cells. By contrast, no appreciable changes in cell viability were observed in the free PPa-treated group, regardless of co-treatment with E64d or excess FA (Fig. 3H). These results indicate that the phototoxicity of FRANT is restored intracellularly through folate receptor–mediated endocytosis followed by lysosomal cathepsin B–mediated cleavage of the di-arginine linker.

**Fig. 4:**
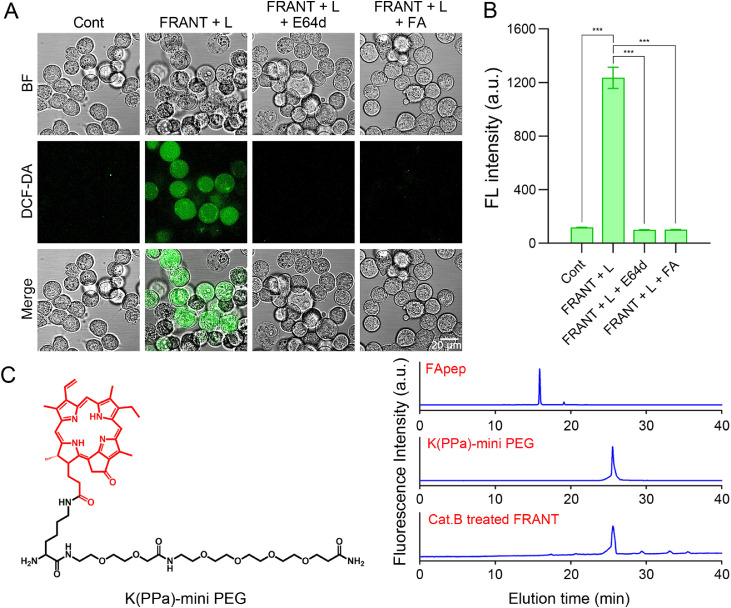
Evaluation of cascade-activatable intracellular ROS generation in LLC-1 cells following FRANT-mediated PDT. (A) Fluorescence images of LLC-1 cells treated with FRANT + L in the presence or absence of the cathepsin B inhibitor E64d or excess FA. Green channel indicates intracellular ROS detected by DCF-DA. Scale bars: 20 μm; (B) Quantitative analysis of intracellular DCF fluorescence intensity (λ_ex._ = 485 nm, λ_em._ = 535 nm). Data are presented as mean ± S.E. Statistical significance was determined using Student’s *t*-test (^⁎⁎⁎^*P* < 0.001); (C) Cathepsin B–mediated cleavage of FApep in FRANT. *Left:* Chemical structure of the FApep cleavage product K(PPa)-miniPEG generated upon cathepsin B treatment. *Right:* HPLC chromatograms of FApep, the cleavage product and cathepsin B–treated FRANT. All samples were analyzed using a fluorescence detector (λ_ex._ = 400 nm, λ_em._ = 663 nm).

We further confirmed that the PDT effect in FRANT-treated cells was attributable to folate receptor– and cathepsin B–mediated intracellular ROS generation during light irradiation (Fig. 4). LLC-1 cells were incubated with FRANT in the presence or absence of each inhibitor, subsequently washed, treated with DCF-DA (a fluorescent ROS probe), and then irradiated with a 670 nm CW laser. Confocal microscopy revealed strong intracellular DCF fluorescence signals in the FRANT-treated group, indicating abundant ROS generation from the cleaved product of FRANT upon light irradiation. As expected, DCF fluorescence intensity was significantly reduced in cells pretreated with either E64d or excess FA, exhibiting 12.38- (*P* *<* 0.001) and 12.23-fold decreases (*P* *<* 0.001), respectively (Fig. 4B).

It is anticipated that the quenched state of FRANT will be selectively dequenched following nanomicelle disassembly after cleavage of the di-arginine linker by cathepsin B in tumors. Therefore, the cleavage of FApep in FRANT by cathepsin B was analyzed. For this purpose, the expected enzymatic cleavage product of FApep, K(PPa)-miniPEG (Fig. 4C), was synthesized and used as a reference. FRANT in sodium acetate buffer (50 mM, pH 5.2) at a PPa-equivalent concentration of 2 µM was treated with 1 unit of cathepsin B at 37 °C for 4 h. Subsequently, FApep, K(PPa)-miniPEG, and cathepsin B-treated FRANT were analyzed by HPLC. FApep and K(PPa)-miniPEG exhibited peak elution times of 18.8 and 25.5 min, respectively. Cathepsin B-treated FRANT displayed a major elution peak at 25.5 min, confirming successful cleavage of FApep in FRANT by cathepsin B.

Taken together, these results demonstrate that the quenched NIR fluorescence and phototoxicity of FRANT can be selectively restored in target cells through folate receptor–mediated endocytosis and cathepsin B activity, thereby enabling tumor cell-specific fluorescence imaging and precisely controlled PDT.

### 3.3 Cascade-activatable induction of ICD in FRANT-treated lung cancer cells

Recent studies have emphasized the immunological properties of PDT *in vivo*, drawing increasing attention to its clinical impact in anticancer therapy. PDT treatment of tumor tissues generates abundant ROS, inducing oxidative stress within tumor cells and leading to extensive cellular damage through apoptotic pathways. Importantly, PDT-induced apoptosis triggers ICD in cancer cells, resulting in the release of damage-associated molecular patterns (DAMPs), including cell-surface exposure of calreticulin and extracellular release of ATP and HMGB1 [36,37]. DAMPs play a critical role in the tumor microenvironment by stimulating immune responses, acting as danger signals for the innate immune system and subsequently activating adaptive immunity, including the maturation and recruitment of cytotoxic CD8^+^ T cells [38]. Meanwhile, avoiding unintended phototoxic damage to normal tissues adjacent to the tumor is essential for confining ICD induction to tumor cells.

**Fig. 5:**
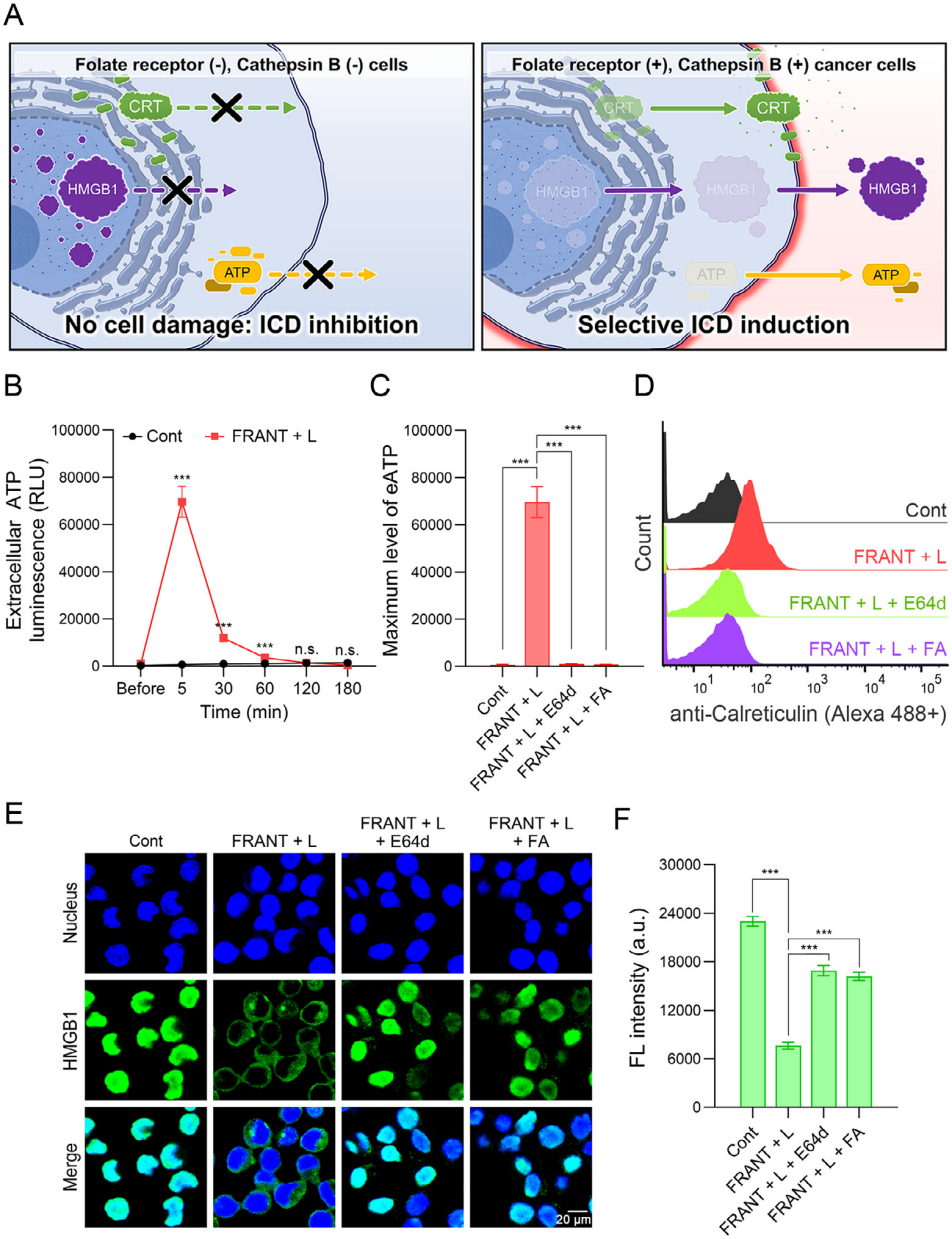
Evaluation of cascade-activatable ICD induction in LLC-1 cells following FRANT-mediated PDT. (A) Schematic overview of *in vitro* experiments for selective ICD induction by FRANT-mediated PDT, dependent on cathepsin B activity or folate receptor expression in cancer cells; (B) Quantitative comparison of eATP release at different time points after FRANT-mediated PDT; (C) Quantitative analysis of eATP release at peak level following FRANT-mediated PDT (*n* = 6 per group); (D) Flow cytometry analysis of calreticulin exposure on cancer cell surfaces following FRANT-mediated PDT; (E) Representative fluorescence images of HMGB1 release following FRANT-mediated PDT; (F) Quantitative analysis of HMGB1 fluorescence intensity from the images in (E). Mean fluorescence intensity was analyzed in >300 cells per group. Data are expressed as mean ± S.E. Statistical significance was determined using Student’s *t*-test (^⁎⁎⁎^*P* < 0.001).

We therefore determined whether FRANT-based PDT selectively induces ICD. LLC-1 cells were subjected to PDT in the presence or absence of E64d and excess FA under identical treatment conditions. Subsequently, key hallmarks of ICD were investigated, including eATP release, membrane exposure of calreticulin, and extranuclear/cellular release of HMGB1 (Fig. 5A).

First, eATP levels rapidly increased at the early time point of 5 min following PDT (FRANT + L, *P* < 0.001) compared with the control group. The levels gradually decreased within 60 min and were barely detectable at 120 and 180 min (Fig. 5B). However, pretreatment with E64d or excess FA (FRANT + L + E64d or FA groups, *P* < 0.001) significantly reduced eATP release compared with the FRANT + L group (Fig. 5C).

Calreticulin exposure was quantified by flow cytometric analysis. Following FRANT-mediated PDT, calreticulin was translocated to and exposed on the membrane surface at 4 h (FRANT + L group, 83.8%). By contrast, the E64d- or excess FA-pretreated groups showed negligible exposure levels (FRANT + L + E64d group, 18.3%; FRANT + L + FA group, 18.4%), compared with the FRANT + L group (Fig. 5D).

Furthermore, the release of nuclear HMGB1 from the cancer cells following FRANT-mediated PDT was examined. After 4 h post-PDT, HMGB1 was largely absent from the nucleus, with only a small amount remaining in the cytoplasm, compared with the control group (3.03-fold decrease, *P* < 0.001). By contrast, pretreatment with E64d (2.21-fold increase, *P* < 0.001) or excess FA (2.12-fold increase, *P* < 0.001) significantly attenuated the extranuclear and cellular release of HMGB1 induced by FRANT-mediated PDT (Fig. 5E and 5F). These results demonstrate that FRANT-mediated PDT selectively triggers cascade-activatable ICD in lung cancer cells. Consequently, its immune-stimulatory properties are expected to synergize with ICI-based immunotherapy, particularly within the tumor microenvironment.

### 3.4 In vivo NIR fluorescence imaging of lung cancer using FRANT

**Fig. 6:**
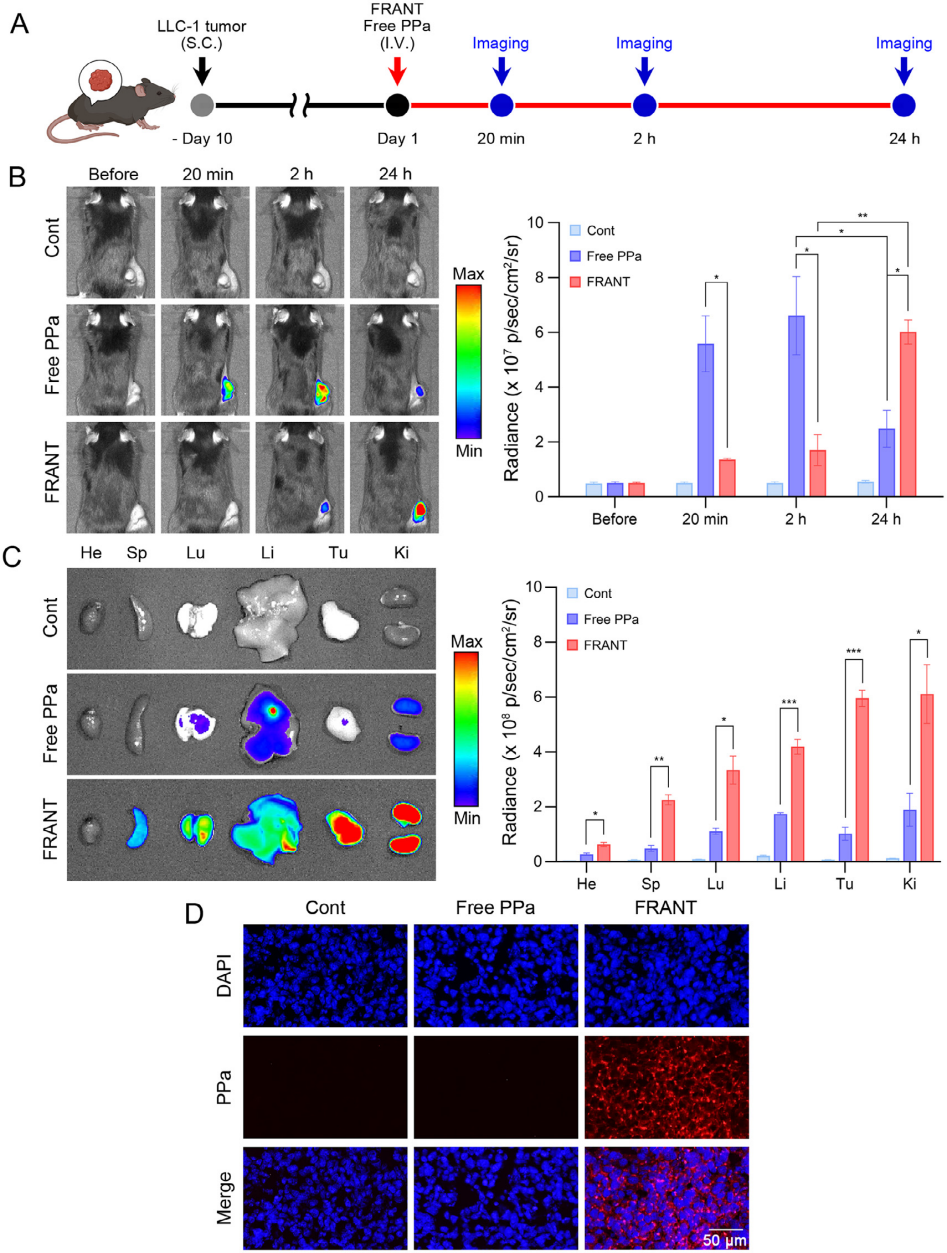
*In vivo* NIR fluorescence imaging of lung cancer using FRANT. (A) Schematic illustration of the experimental timeline for *in vivo* NIR fluorescence imaging of free PPa and FRANT; (B) NIR fluorescence images of LLC-1 tumor-bearing C57BL/6 mice at different time points following injection of free PPa or FRANT (left), with quantitative analysis of tumor-site NIR fluorescence intensity (right, *n* = 3 per group); (C) *Ex vivo* NIR fluorescence images of tumors and major organs collected 24 h post-injection (left). Organs shown: heart (He), spleen (Sp), lung (Lu), liver (Li), tumor (Tu) and kidney (Ki). Quantitative analysis of NIR fluorescence intensity in tumors and major organs is shown (right); (D) Confocal fluorescence images of tumor sections from the control, free PPa and FRANT groups. Cell nuclei were counterstained with DAPI. Scale bars: 50 μm. Data are expressed as mean ± S.E. Statistical significance was determined using Student’s *t*-test (*P < 0.05, ^⁎⁎^*P* < 0.01, ^⁎⁎⁎^*P* < 0.001).

The ability of FRANT to selectively detect cancer sites is critical for imaging-guided PDT. To assess this capability, *in vivo* NIR fluorescence imaging was performed in an LLC-1 lung tumor model. Tumor-bearing mice were intravenously administered either free PPa or FRANT at a PPa dose of 5 mg/kg. Fluorescence imaging was conducted at 20 min, 2 h and 24 h post-injection (Fig. 6A). A control group received an intravenous injection of saline.

Mice injected with free PPa exhibited strong fluorescence signals at tumor sites at 20 min and 2 h post-injection; however, substantial fluorescence was also detected in surrounding normal tissues, indicating nonspecific accumulation of free PPa in the skin (Figs. 6B and S3). At 24 h post-injection, the fluorescence intensity at tumor sites in free PPa-treated mice was markedly reduced (2.66-fold decrease, *P* < 0.05) compared with that at 2 h, which may be attributed to the efflux of PPa from the cancer cells (Fig. 3B). By contrast, FRANT-treated mice displayed significantly lower tumor fluorescence signals than the free PPa group at 20 min and 2 h post-injection (*P* < 0.05), consistent with the maintenance of fluorescence quenching at these time points (Fig. 3B).

Notably, the fluorescence intensity of FRANT reached its maximum at 24 h, clearly delineating tumor sites and margins in the NIR fluorescence images. Consistent with the *in vitro* cell studies (Fig. 3, Fig. 4), tumor-confined fluorescence dequenching indicates that cascade-activated recovery of ROS generation and PDT efficacy in tumor cells can be achieved *in vivo* using FRANT. Collectively, these results provide strong evidence for tumor-specific fluorescence dequenching by FRANT compared with free PPa.

Subsequently, *ex vivo* fluorescence imaging of major organs (i.e., heart, spleen, lung, liver and kidney) and tumors was performed at 24 h post-injection (Fig. 6C). The free PPa group showed weak fluorescence in these tissues, suggesting that free PPA was largely cleared from the body and did not accumulate appreciably in tumors. By contrast, the FRANT group exhibited strong fluorescence signals in tumors.

Confocal fluorescence imaging of tumor sections further confirmed the preferential uptake and strong fluorescence generation of FRANT in tumor tissues, compared with those in free PPa-treated mice (Fig. 6D). Together with the data in Fig. 5D, these results indicate that FRANT preferentially accumulates in folate receptor-overexpressing tumors via both the enhanced permeability and retention effect [39] and folate receptor–mediated endocytosis, followed cathepsin B–mediated fluorescence recovery within tumor cells. These findings demonstrate the potential of FRANT for precision imaging-guided PDT of folate receptor-overexpressing lung cancers *in vivo*.

In *ex vivo* NIR fluorescence imaging, accumulation of FRANT was also detected in kidney tissues (Fig. 6C), attributable to the overexpression of folate receptors in renal cells [40]. Folate receptor expression in non-target organs such as the kidney has been recognized as a limitation of FA-conjugated nanomedicine [41,42]. However, FRANT exhibited no cytotoxicity even at high concentrations in the absence of light. Moreover, because the lung is anatomically distant from the kidney, light irradiation directed at lung tissues is unlikely to induce renal damage, in contrast to conventional cytotoxic chemotherapeutic agents.

The additional biodistribution experiments were conducted using tumor-free mice to evaluate the temporal changes in NIR fluorescence intensities in the blood and major organs of FRANT-treated mice up to 72 h post-injection (in Supplementary Information). The results showed that PPa fluorescence signals in the blood continuously decreased over 72 h and were reduced to levels comparable to those of control mice without FRANT injection at 72 h post-injection (Fig. S4), indicating systemic clearance of FRANT. Among the examined organs, the heart, lungs, spleen, liver and kidneys showed the highest PPa fluorescence signals at 24 h post-injection, and the fluorescence signals from these organs were markedly reduced at 72 h post-injection (Fig. S5).

### 3.5 Antitumor effects of cascade-activatable FRANT in combined PDT and immunotherapy

**Fig. 7:**
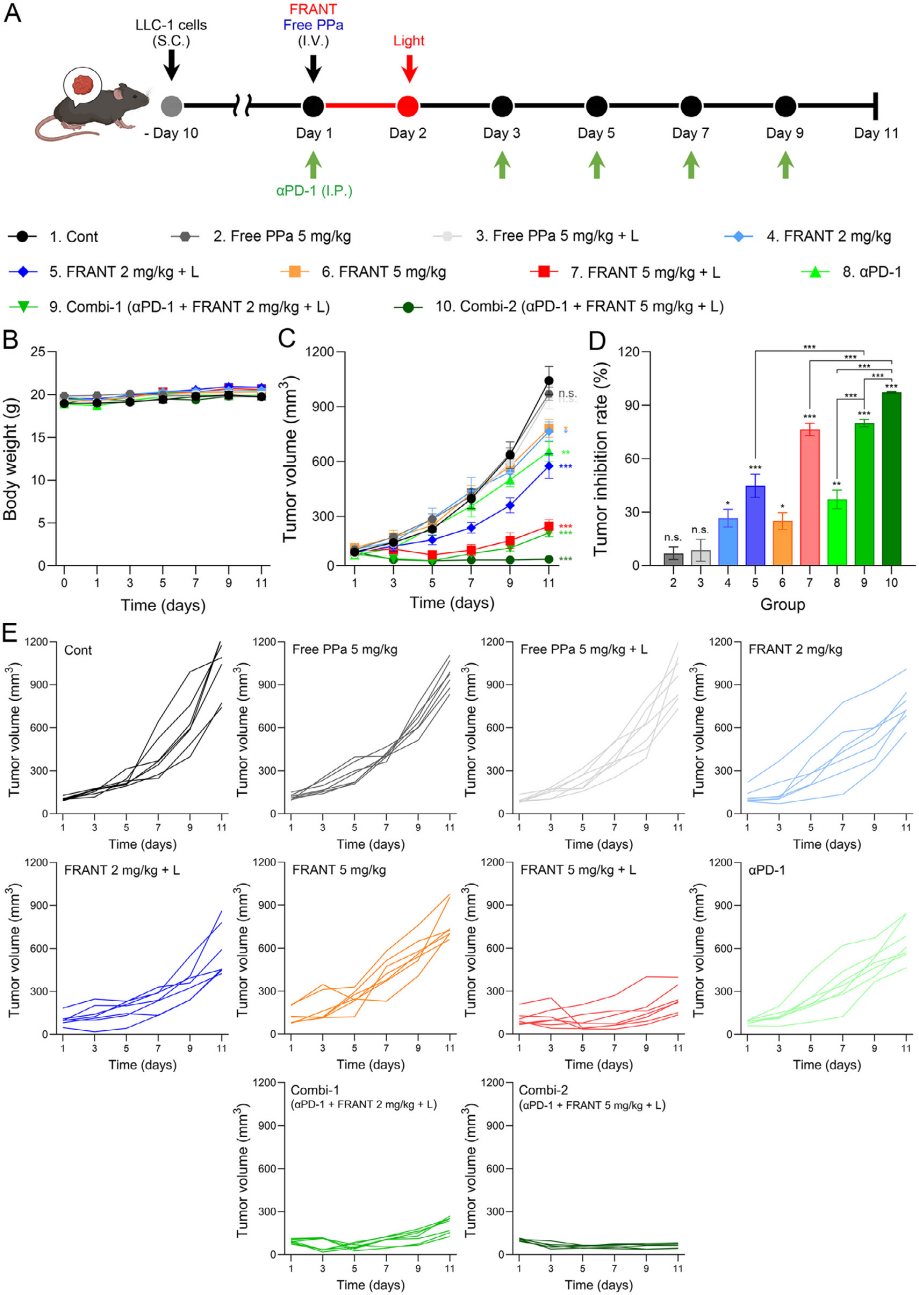
Antitumor efficacy of FRANT-mediated PDT combined with immune checkpoint blockade. (A) *In vivo* experimental schedule for evaluating the antitumor efficacy of FRANT-mediated PDT and combination therapy with an αPD-1 antibody; (B) Body weight changes in each treatment group over time; (C) Average tumor growth kinetics of mice in each treatment group (*n* = 7 per group); (D) Average tumor inhibition rate (%) at the experimental endpoint (Day 11); (E) Individual tumor growth curves for mice in each group. All data are expressed as mean ± S.E. Statistical significance was determined using Student’s *t*-test (*P < 0.05, ^⁎⁎^*P* < 0.01, ^⁎⁎⁎^*P* < 0.001).

The *in vivo* PDT efficacy of FRANT, either as monotherapy or in combination with an ICI, was evaluated by monitoring tumor growth following each treatment. The LLC-1 tumor model is well known to form an immune-evasive “cold” tumor microenvironment that often limits the immunological benefits of PDT and immunotherapy [43,44]. LLC-1 lung cancer cells were subcutaneously implanted into the dorsal flanks of C57BL/6 mice. When tumor volumes reached ∼ 100 mm^3^, the mice were randomly divided into ten groups (*n* = 7 per group). On Day 1, tumor-bearing mice received an intravenous injection of FRANT (2 or 5 mg/kg) or free PPa (5 mg/kg). PDT was performed on Day 2 by irradiating the tumor sites with a 670 nm CW laser. For immunotherapy, αPD-1 (10 mg/kg) was administered intraperitoneally every 2 d from Day 1 to Day 9 (Fig. 7A).

No appreciable differences in body weight were observed among the treatment groups compared with the control group (Fig. 7B). The therapeutic efficacy against LLC-1 tumors in each treatment group was evaluated based on average tumor growth kinetics (Fig. 7C) and inhibition rates at the experimental endpoint (Fig. 7D). Tumor volumes in free PPa-treated mice (Groups 2 and 3) remained comparable to those in the control group, regardless of laser irradiation (*P* > 0.05). Mice in the FRANT-treated groups without laser irradiation (Groups 4 and 6) exhibited moderate tumor growth inhibition: 26.6% for the 2 mg/kg dose (*P* < 0.05) and 25.1% for the 5 mg/kg dose (*P* < 0.05) on Dday 11. As expected, mice in the FRANT + L groups (Groups 5 and 7) demonstrated significantly enhanced, dose-dependent tumor growth inhibition—44.8% in Group 5 and 76.4% in Group 7—compared with the control group. Monotherapy with αPD-1 (Group 8) resulted in 37.2% tumor growth inhibition. Notably, combination therapy with αPD-1 and FRANT + L (Groups 9 and 10) produced a synergistic antitumor effect, with 79.9% and 97.3% reductions in tumor growth relative to the control, respectively. The CDI values for Groups 9 and 10 were 0.58 and 0.18, respectively, indicating a significantly synergistic enhancement of the antitumor effect achieved by combining FRANT-mediated PDT and immunotherapy.

**Fig. 8:**
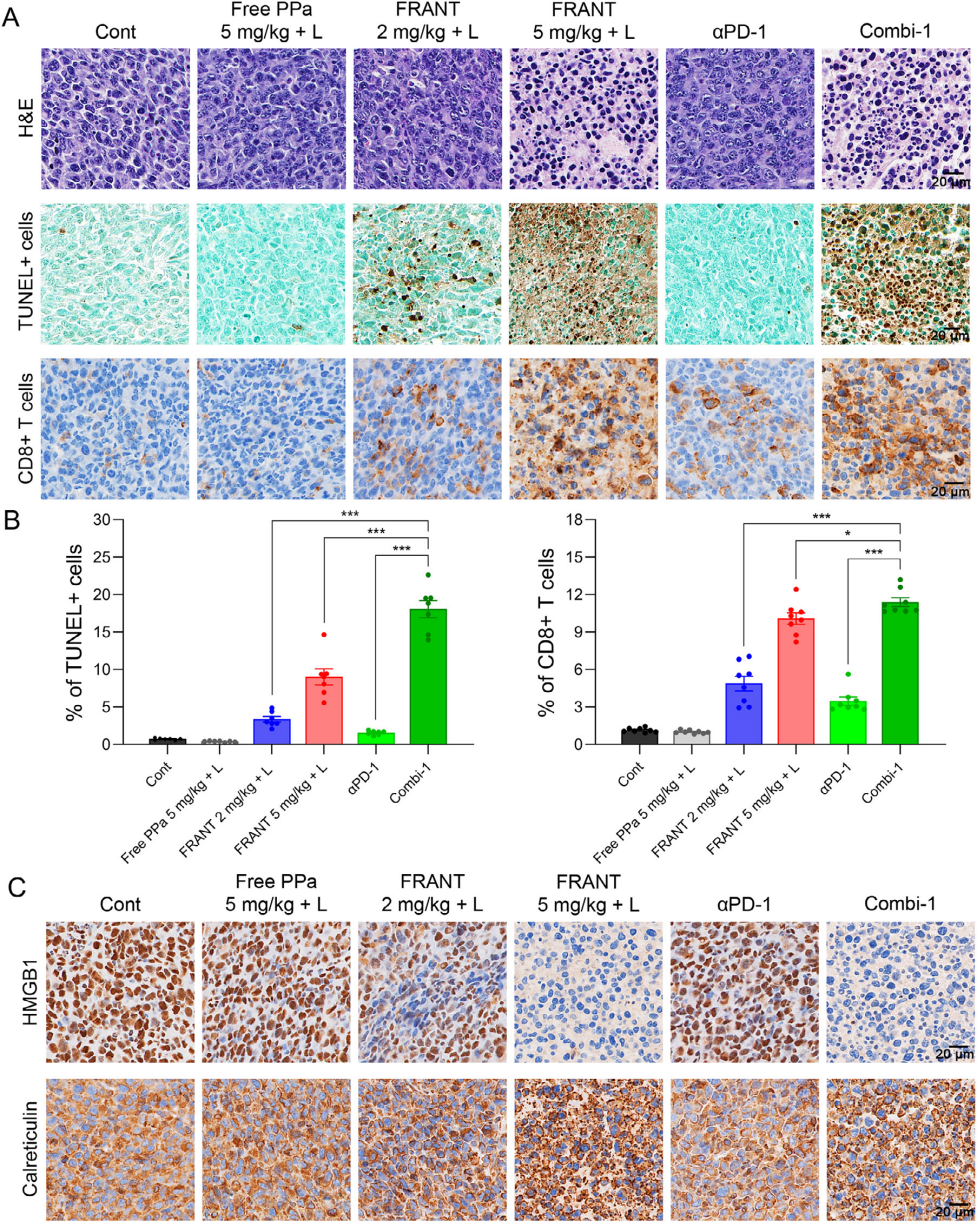
Histological analysis of LLC-1 tumors. (A) First panel: representative images of H&E-stained sections of LLC-1 tumors collected 24 h after light irradiation (Day 3). Second panel: representative TUNEL-stained tumor sections collected on Day 3. Third panel: representative IHC images of CD8^+^ T cells in tumor sections collected on Day 3. Scale bars: 20 μm; (B) Quantitative analysis of TUNEL^+^ cancer cells (left) and CD8^+^ T cells (right). Group 10 (Combi-2) was excluded from these analyses owing to insufficient tumor size for tissue sectioning; (C) IHC images of HMGB1 (upper panel) and calreticulin (lower panel) in tumor sections collected on Day 3. Scale bars: 20 μm. Data are expressed as mean ± S.E. Statistical significance was determined using Student’s *t*-test (*P < 0.05, ^⁎⁎⁎^*P* < 0.001).

In our *in vivo* study (Fig. 7C), the Free PPa + L group exhibited tumor growth kinetics virtually identical to those of the control group. Moreover, tumor tissue analyses demonstrated that apoptotic cell death, CD8⁺ T cell infiltration, and ICD markers (calreticulin exposure and HMGB1 release) in the Free PPa + L group were indistinguishable from control levels (Fig. 8). These findings indicate that free PPa–mediated PDT did not induce measurable antitumor immune activation under the experimental conditions of this study.

A modest tumor growth delay was observed in FRANT-treated mice without light irradiation, emerging after day 9. Although FRANT exhibited negligible dark cytotoxicity *in vitro*, the *in vivo* tumor microenvironment involves complex cellular and stromal interactions that may contribute to delayed growth modulation. The underlying mechanism warrants further investigation.

### 3.6 Analysis of in vivo ICD and enhanced immune cell infiltration

To further evaluate the antitumor efficacy of FRANT in terms of *in vivo* ICD induction and enhanced immune cell infiltration, an additional cohort of tumor-bearing mice was prepared. The mice received the same treatment regimen as described above. On Day 3, tumor tissues from all groups were collected, sectioned and stained. As the tumor sizes in Group 10 were too small on Day 3 to allow tissue sectioning, this group was excluded. H&E staining of tumor sections in Groups 7 and 9 revealed large number of small-sized tumor cells with condensed nuclei and infiltrating inflammatory cells (Figs. 8A and S6). TUNEL staining was performed to investigate apoptotic cell death in the tumors (Figs. 8A and S7). The number of apoptotic tumor cells was 4.55-fold higher in the FRANT 2 mg/kg + L group (*P* < 0.001) and 12.2-fold higher in the FRANT 5 mg/kg + L group (*P* < 0.001) than in the control group. As expected, combined treatment of αPD-1 and FRANT 2 mg/kg + L (Group 9) significantly increased the number of apoptotic cells in LLC-1 tumors compared with both the FRANT 2 mg/kg + L group (5.38-fold increase, *P* < 0.001) and the FRANT 5 mg/kg + L group (2.0-fold increase, *P* < 0.001) (Fig. 8B).

Furthermore, immunohistochemistry (IHC) staining revealed CD8-positive cells indicative of cytotoxic T cell infiltration in all therapy groups (Figs. 8A and S8). Compared with the control, the FRANT 2 mg/kg + L group (Group 5) exhibited a 4.25-fold increase in CD8^+^ T cell infiltration (*P* < 0.001), whereas the FRANT 5 mg/kg + L group (Group 7) showed an 8.78-fold increase (*P* < 0.001). Although αPD-1 monotherapy (Group 8) induced a relatively modest effect (3.02-fold increase), combined treatment with αPD-1 and FRANT 2 mg/kg + L (Group 9) resulted in markedly elevated infiltration, reaching a 9.92-fold increase compared with the control group (*P* < 0.001). This combination therapy also showed significantly greater infiltration compared with both the 2 mg/kg + L group (*P* < 0.001) and the FRANT 5 mg/kg + L group (*P* < 0.05) (Fig. 8B).

To corroborate these findings, we investigated the expression of ICD-related proteins in LLC-1 tumor cells. FRANT-mediated PDT induced a dose-dependent reduction in nuclear HMGB1 expression compared with the control group (Figs. 8C and S9). Additionally, damaged tumor cells exhibited cell-surface exposure of calreticulin following treatment, consistent with its translocation from the cytoplasm to the plasma membrane (Figs. 8C and S10).

In our *in vivo* study, FRANT-mediated PDT induced significant apoptosis and ICD in tumor cells 24 h post-PDT (Figs. 8 and S7–S10). Importantly, HMGB1 release and calreticulin exposure were clearly observed in the FRANT-mediated PDT groups, providing histological evidence of ICD triggered by photodynamic damage (Figs. 8C and S9–S10). These events markedly increased the infiltration of CD8^+^ T cells and other inflammatory cells (Figs. 8 and S6&S8). Collectively, these processes promote host antitumor immunity through DAMP emission, serving as a starting point for converting immune-cold LLC-1 tumors into an immune-hot state.

Furthermore, histological analysis of tumors from the combination treatment group revealed the highest CD8^+^ T cell infiltration compared with the monotherapy groups, indicating enhanced involvement of cytotoxic T cells in the antitumor response (Figs. 8A, 8B and S8). These results provide strong evidence that FRANT-based PDT acts as a catalyst for initiating antitumor immune responses within the tumor, thereby substantially enhancing immunotherapeutic efficacy.

Taken together, these results demonstrate that FRANT-mediated PDT exerts potent antitumor efficacy through apoptosis and ICD induction, effectively converting immune-cold LLC-1 tumors into immunologically hot tumors and thereby maximizing the therapeutic benefit of combination treatment with αPD-1.

### 3.7 Safety of FRANT and treatment regimen in vivo

**Fig. 9:**
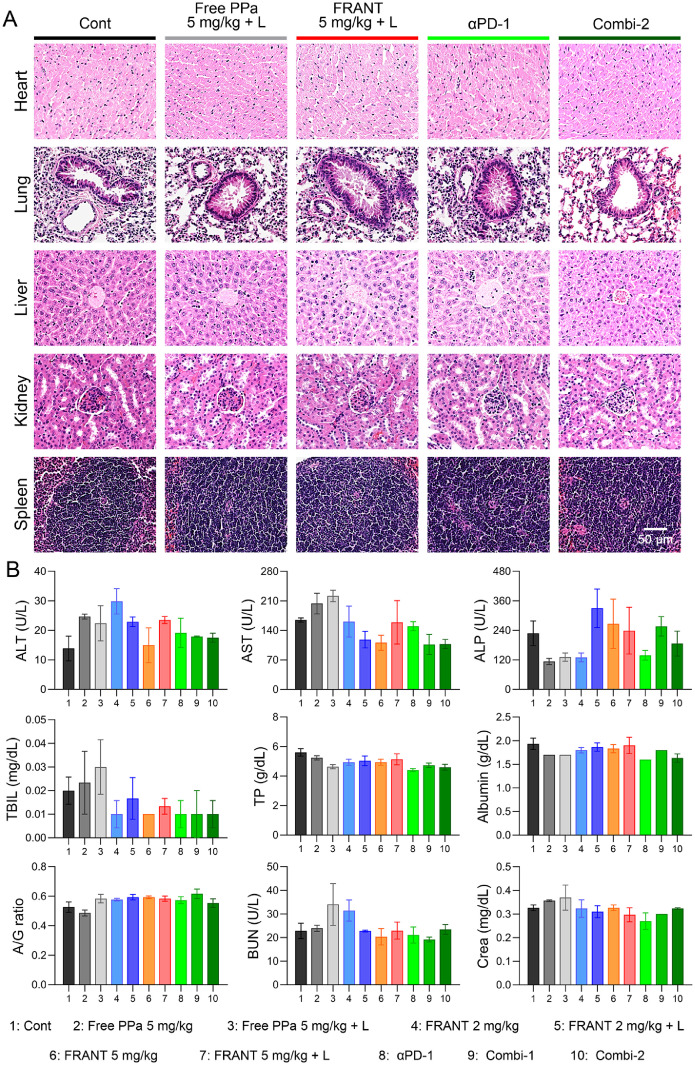
Safety of FRANT and treatment regimen *in vivo.* (A) Histopathological images of major organs collected on Day 10 following PDT and combination therapy. Scale bars: 50 μm; (B) Serum biochemistry analysis of blood samples collected on Day 10 following PDT and combination therapy.

We evaluated whether FRANT-mediated PDT, either as monotherapy or in combination with immune checkpoint blockade, induces toxicity to normal cells or organs. Major organs (i.e., heart, lung, kidney, spleen and liver) and blood samples were collected from each treatment group on Day 10. H&E staining of tissue sections revealed no apparent histopathological changes in any of the treatment groups (Figs. 9A and S11). Serum biochemical analysis showed that blood biomarker levels, including those indicative of liver and kidney function, remained within normal ranges across all treatment groups compared with the control (Fig. 9B). These results confirm the biocompatibility of FRANT and the safety of all treatment regimens under the experimental conditions.

In terms of clinical translation, one of the major challenges commonly encountered in nanomedicine development is the feasibility of large-scale synthesis and manufacturing under clinically relevant conditions. In the case of FRANT, however, the materials used and the conjugation strategy are based on well-established chemical processes, suggesting that large-scale production may be feasible without substantial technical barriers. Another important consideration is tumor heterogeneity, particularly the variability in target receptor expression across different tumor types and individual patients. Because the therapeutic mechanism of FRANT relies on folate receptor–mediated targeting, differences in folate receptor expression levels could potentially influence treatment efficacy. Nevertheless, folate receptors are known to be highly expressed in several epithelial malignancies while showing relatively limited expression in most normal tissues [41,42], which has made them attractive targets for tumor-selective drug delivery strategies. In addition to the tumor model investigated in this study, folate receptor overexpression has been reported in several other cancer types, including ovarian cancer, gastrointestinal cancers, breast cancer (including triple-negative breast cancer), and colon cancer [41,42]. Therefore, these tumor types may also represent potential therapeutic targets for the currently developed FRANT platform, although further investigation in diverse tumor models will be necessary to fully validate its broader clinical applicability.

## 4 Conclusion

In summary, we developed a cascade-activatable small-molecule nanotheranostic micelle (FRANT) that enables serum-stable quenching and tumor-selective activation via folate receptor–mediated uptake and cathepsin B–triggered cleavage. FRANT achieved precise NIR fluorescence imaging and PDT of lung cancer while also inducing cascade-activatable ICD in tumor cells. Notably, when combined with PD-1 blockade, FRANT-mediated PDT converted immune-cold tumors into immune-active ones, eliciting robust CD8^+^ T cell infiltration and durable tumor regression without detectable systemic toxicity. Collectively, these findings position FRANT as a promising theranostic platform that integrates diagnostic precision with immunomodulatory efficacy, offering a powerful strategy for next-generation lung cancer treatment.

## Conflicts of interest

The authors declare that there is no conflicts of interest.

## Appendix Supplementary materials

Image, application 1

## References

[1] Sung H., Ferlay J., Siegel R.L., Laversanne M., Soerjomataram I., Jemal A. (2021). Global cancer statistics 2020: GLOBOCAN estimates of incidence and mortality worldwide for 36 cancers in 185 countries. CA Cancer J Clin.

[2] Bray F., Laversanne M., Sung H., Ferlay J., Siegel R.L., Soerjomataram I. (2024). Global cancer statistics 2022: GLOBOCAN estimates of incidence and mortality worldwide for 36 cancers in 185 countires. CA Cancer J Clin.

[3] Feng J., Zhang P., Wang D., Li Y., Tan J. (2024). New strategies of lung cancer diagnosis and treatment: applications and advances in nanotechnology. Biomark Res.

[4] Reck M., Rodríguez-Abreu D., Robinson A.G., Hui R., Csöszi T., Fülöp A. (2016). Pembrolizumab versus chemotherapy for PD-L1-positive non-small-cell lung cancer. N Engl J Med.

[5] Gandhi L., Rodríguez-Abreu D., Gadgeel S., Esteban E., Felip E., Angelis F. (2018). Pembrolizumab plus chemotherapy in metastatic non-small-cell lung cancer. N Engl J Med.

[6] Katsurada M., Nagano T., Tachihara M., Kiriu T., Furukawa K., Koyama K. (2019). Nishimura, baseline tumor size as a predictive and prognostic factor of immune checkpoint inhibitor therapy for non-small cell lung cancer. Anticancer Res.

[7] Huang J., Theelen W.S.M.E., Belcaid Z., Najjar M., Geest D., Singh D. (2025). Combination of pembrolizumab and radiotherapy induces systemic antitumor immune response in immunologically cold non-small cell lung cancer. Nat Cancer.

[8] Huang M., Lou Y., Pellissier J., Burke T., Liu F.X., Xu R. (2017). Cost effectiveness of pembrolizumab vs. standard-of-care chemotherapy as first-line treatment for metastatic NSCLC that expresses high levels of PD-L1 in the United States. Pharmacoeconomics.

[9] Johnson D.B., Nebhan C.A., Moslehi J.J., Balko J.M. (2022). Immune-checkpoint inhibitors: long-term implications of toxicity. Nat Rev Clin Oncol.

[10] Pham T.C., Nguyen V.N., Choi Y., Lee S., Yoon J. (2021). Recent strategies to develop innovative photosensitizers for enhanced photodynamic therapy. Chem Rev.

[11] Kim D., Lee S., Na K. (2021). Immune stimulating antibody-photosensitizer conjugates via Fc-mediated dendritic cell phagocytosis and phototriggered immunogenic cell death for KRAS-mutated pancreatic cancer. Small.

[12] Showalter A., Limaye A., Oyer J.L., Igarashi R., Kittipatarin C., Copik A.J. (2017). Cytokines in immunogenic cell death: applications for cancer immunotherapy. Cytokine.

[13] Turubanova V.D., Balalaeva I.V., Mishchenko T.A., Catanzaro E., Alzeibak R., Peskova N.N. (2019). Immunogenic cell death induced by a new photodynamic therapy based on photosens and photodithazine. J Immunother Cancer.

[14] Kwon N., Weng H., Rajora M.A., Zheng M.R.G. (2025). Activatable photosensitizers: from fundamental principles to advanced designs. Angew Chem Int Ed.

[15] Brown S.B., Brown E.A., Walker I. (2004). The present and future role of photodynamic therapy in cancer treatment. Lancet Oncol.

[16] Li Y., Lee H., Go E.M., Lee S.S., Han C., Choi Y. (2024). Strongly quenched activatable theranostic nanogel for precision imaging-guided photodynamic therapy and enhanced immunotherapy. J Control Release.

[17] Lovell J.F., Jin C.S., Huynh E., Jin H., Kim C., Rubinstein J.L. (2011). Porphysome nanovesicles generated by porphyrin bilayers for use as multimodal biophotonic contrast agents. Nat Mater.

[18] Kim J., Won Y., Goh S.H., Choi Y. (2016). A redox-responsive theranostic agent for target-specific fluorescence imaging and photodynamic therapy of EGFR-overexpressing triple-negative breast cancers. J Mater Chem B.

[19] Tam L.K.B., He L., Ng D.K., Cheung C.K., Lo P.C. (2022). A tumor-targeting dual-stimuli-activatable photodynamic molecular beacon for precise photodynamic therapy. Chem A Eur J.

[20] Nunez M.I., Behrens C., Woods D.M., Lin H., Suraokar M., Kadara H. (2012). High expression of folate receptor alpha in lung cancer correlates with adenocarcinoma histology and mutation. J Thorac Oncol.

[21] Gong F., Peng X., Luo C., Shen G., Zhao C., Zou L. (2013). Cathepsin B as a potential prognostic and therapeutic marker for human lung squamous cell carcinoma. Mol Cancer.

[22] Xu J., Song M., Fang Z., Zheng L., Huang X., Liu K. (2023). Applications and challenges of ultra-small particle size nanoparticles in tumor therapy. J Control Release.

[23] Stylianopoulos T., Jain R.K. (2015). Design considerations for nanotherapeutics in oncology. Nanomedicine.

[24] Islam M.A., Barua S., Barua D. (2017). A multiscale modeling study of particle size effects on the tissue penetration efficacy of drug-delivery nanoparticles. BMC Syts Biol.

[25] Li C., Guan H., Li Z., Wang F., Wu J., Zhang B. (2020). Study on different particle sizes of DOX-loaded mixed micelles for cancer therapy. Colloids Surf B Biointerfaces.

[26] Savellano M.D., Owusu-Brackett N., Son J., Ganga T., Leung N.L., Savellano D.H. (2013). Photodynamic tumor eradication with a novel targetable photosensitizer: strong vascular effects and dependence on treatment repetition versus potentiation. Photochem Photobiol.

[27] Shin M., Choi Y.E., Li Y., Goh S.H., Choi Y. (2023). FOXM1 inhibitor-loaded nanoliposomes for enhanced immunotherapy against cancer. Chem Eng J.

[28] Myrzakhmetov B., Arnoux P., Mordon S., Acherar S., Tsoy I., Frochot C. (2021). Photophysical properties of protoporphyrin IX, pyropheophorbide-a and photofrin® in different conditions. Pharmaceuticals.

[29] He Y.Y., Wang X.C., Jin P.K., Zhao B., Fan X. (2009). Complexation of anthracene with folic acid studied by FTIR and UV spectroscopies. Spectrochim Acta A Mol Biomol Spectrosc.

[30] Bhatti M., Yahioglu G., Milgrom L.R., Garcia-Maya M., Chester K.A., Deonarain M.P. (2008). Targeted photodynamic therapy with multiply-loaded recombinant antibody fragments. Int J Cancer.

[31] Gao H., Shi W., Freund L.B. (2005). Mechanics of receptor-mediated endocytosis. PNAS.

[32] Kang H., Rho S., Stiles W.R., Hu S., Beak Y., Hwang D.W. (2020). Size-dependent EPR effect of polymeric nanoparticles on tumor targeting. Adv Healthc Mater.

[33] Choi H.S., Liu W., Misra P., Tanaka E., Zimmer J.P., Ipe B.I. (2007). Renal clearance of quantum dots. Nat Biotechnol.

[34] DeRosa M.C., Crutchley R.J. (2002). Photosensitized singlet oxygen and its applications. Coord Chem Rev.

[35] Gondi C.S., Rao J.S. (2013). Cathepsin B as a cancer target. Expert Opin Ther Targets.

[36] Aebisher D., Woznicki P., Bartusik-Aebisher D. (2024). Photodynamic therapy and adaptive immunity induced by reactive oxygen species. Recent Rep Cancers.

[37] Wang N., Zhao Z., Xiao X., Mo L., Yao W., Yang H. (2023). ROS-responsive self-activatable photosensitizing agent for photodynamic-immunotherapy of cancer. Acta Biomater.

[38] Reginato E., Wolf P., Hamblin M.R. (2014). Immune response after photodyanmic therapy increases anti-cancer and anti-bacterial effects. World J Immunol.

[39] Shinde V.R., Revi N., Murugappan S., Singh S.P., Rengan A.K. (2022). Enhanced permeability and retention effect: a key facilitator for solid tumor targeting by nanoparticles. Photodiagnosis Photodyn Ther.

[40] Knight S.F., Kundu K., Joseph G., Dikalov S., Weiss D., Murthy N. (2012). Folate receptor-targeted antioxidant therapy ameliorates renal ischemia-reperfusion injury. J Am Soc Nephrol.

[41] Parker N., Turk M.J., Westrick E., Lewis J.D., Low P.S., Leamon C.P. (2005). Folate receptor expression in carcinomas and normal tissues determined by a quantitative radioligand binding assay. Anal Biochem.

[42] Shakeri-Zadeh A., Rezaeyan A., Sarikhani A., Ghaffari H., Samadian H., Khademi S. (2021). Folate receptor-targeted nanoprobes for molecular imaging of cancer: friend or foe?. Nanotoday.

[43] Sun C., Nagaoka K., Kobayashi Y., Nakagawa H., Kakimi K., Nakajima J. (2021). Neoantigen dendritic cell vaccination combined with anti-CD38 and CpG elicits anti-tumor immunity against the immune checkpoint therapy-resistant murin lung cancer cell line LLC1. Cancers (Basel).

[44] Dudzik T., Domanski I., Makuch S. (2024). The impact of photodynamic therapy on immune system in cancer – an update. Front Immunol.

