# Supplementary material for: Cascade-activatable small-molecule theranostic nanomicelles for photodynamic-immunotherapy of immune-cold lung tumors
Source: Asian J Pharm Sci. 2026 Apr 29;21(3):101152. doi: 10.1016/j.ajps.2026.101152 (PMC13261983; doi:10.1016/j.ajps.2026.101152)
Supplement: Supplementary file 1 [file mmc1.docx]

Supplementary Information

**Cascade-Activatable Small-Molecule Theranostic Nanomicelles for Photodynamic-Immunotherapy of Immune-Cold Lung Tumors**

Young-Chan Yoon^#^, Hyung-Jun Kim^#^, and Yongdoo Choi*^1^

Division of Technology Convergence, National Cancer Center, 323 Ilsan-ro, Goyang, Gyeonggi-Do 10408, Republic of Korea.

^#^ These authors contributed equally to this work.

* Corresponding author. E-mail: [ydchoi@ncc.re.kr](mailto:ydchoi@ncc.re.kr) (Y. Choi)

**Methods**

**1. Quantification of FApep and F68 in FRANT**

The content of FApep and F68 in FRANT was quantified using a UV–Vis spectrophotometer.

To determine the concentration of FApep in FRANT, a stock solution of PPa was prepared by dissolving 1 mg of PPa in 1 mL of DMSO. PPa standard solutions were then prepared by serial dilution to obtain concentrations ranging from 19 μM to 0.19 μM. The absorbance of the standard solutions was measured at 666 nm to determine the molar extinction coefficient of PPa. FRANT was dissolved in DMSO, and the absorbance of the resulting solution was measured at 666 nm. The concentration of FApep was calculated using the molar extinction coefficient of PPa because the molar amounts of PPa and FApep are identical.

The concentration of F68 in FRANT was determined using the cobalt thiocyanate method with slight modifications [1]. The cobalt thiocyanate solution was prepared by dissolving 0.3 g of cobalt nitrate hexahydrate (Sigma, USA) and 1.2 g of ammonium thiocyanate (Sigma, USA) in 3 mL of distilled water. For the preparation of standard solutions, a 2% F68 solution was serially diluted to concentrations ranging from 1.5% to 0.2%. Subsequently, cobalt thiocyanate solution (100 μL) was mixed with ethyl acetate (200 μL), ethanol (80 μL), and the sample solution (F68 solution or FRANT solution, 40 μL). The mixture was centrifuged at 14,000 rpm for 1 min, and the precipitate was washed with ethyl acetate until the supernatant became clear. After air-drying the precipitate, it was dissolved in 1 mL of acetone. The absorbance of the cobalt thiocyanate complex formed with F68 or FRANT was measured at 623 nm. The concentration of F68 in FRANT was calculated using the calibration curve obtained from the F68 standard solutions. Finally, the molar ratio of FApep to F68 in FRANT was determined based on the quantified amounts of FApep and F68.

**Reference**

[1] Chung HH, Zhou, Khor HK, Qiu J. Direct determination of residual Pluronic F-68 in in-process samples from monoclonal antibody preparations by high performance liquid chromatography. J Chromatogr A 2011;1218: 2106-13.

**Results**

Table S1. Determination of FApep and F68 contents in FRANT.

|  | **FApep** | **F68** |
| --- | --- | --- |
| **Molar ratio** | **1** | **0.07** |

**2. Flow Cytometry**

Basal expression levels of folate receptor and cathepsin B in LLC-1 cells were analyzed by flow cytometry.

To measure surface folate receptor levels, LLC-1 cells were suspended in cold FACS buffer (Invitrogen, CA, USA) and adjusted to a final concentration of 0.5–1 × 10^6^ cells. After centrifugation and removal of the supernatant, the cells were incubated with an anti-folate receptor α primary antibody (1:25, Invitrogen) at 25 °C for 30 min. Following two washes by centrifugation at 1,500 rpm for 5 min, the cells were incubated in the dark with an Alexa Fluor 488-conjugated anti-rabbit secondary antibody (1:50, Abcam, Cambridge, UK) at 25 °C for 30 min. The stained cells were analyzed using a FACSLyric II flow cytometer (BD Biosciences, NJ, USA).

To measure intracellular cathepsin B levels, LLC-1 cells were washed twice with DPBS and adjusted to a density of 1 × 10^6^ cells. After centrifugation and removal of the supernatant, cell pellets were fixed with 4% paraformaldehyde (T&I Biotechnology, Chuncheon, Korea) for 20 min on ice. The fixed cells were then washed twice and permeabilized with 0.1% Tween 20 at 25 °C for 20 min. To block non-specific antibody binding, the cells were incubated with 3% bovine serum albumin (GenDEPOT, TX, USA) at 25 °C for 1 h. After two additional washes, the cells were incubated with an anti-cathepsin B primary antibody (1:50, Cell Signaling Technology, MA, USA) at 25 °C for 1 h. Subsequently, the cells were washed and incubated in the dark with an Alexa Fluor 488-conjugated anti-rabbit secondary antibody (1:100) at 25 °C for 40 min. The stained cells were analyzed using a FACSLyric II flow cytometer.

**3. Biodistribution study using tumor-free mice**

To examine the biodistribution of FRANT in vivo over an extended time period, a total of fifteen female C57BL/6 mice were used. Three mice in the control group received an intravenous injection of saline and were sacrificed 5 min later. Blood samples and major organs (heart, lungs, spleen, liver, and kidneys) were collected. *Ex vivo* fluorescence imaging (λ_ex._ = 620/20 nm, λ_em._ = 710/40 nm) of serum obtained from the blood samples and the collected organs was performed using an IVIS Lumina XRMS system (Xenogen Corporation–Caliper, CA, USA).

Twelve mice in the FRANT-treated groups received an intravenous injection of FRANT at a dose of 5 mg PPa equivalent per kg body weight. The mice were sacrificed at 5 min (*n* = 3), 24 h (*n* = 3), 48 h (*n* = 3), and 72 h (*n* = 3) post-injection. Blood samples and major organs (heart, lungs, spleen, liver, and kidneys) were collected. *Ex vivo* fluorescence imaging (λ_ex._ = 620/20 nm, λ_em._ = 710/40 nm) of serum obtained from the blood samples and the collected organs was performed using the IVIS Lumina XRMS system.

**Results**

**
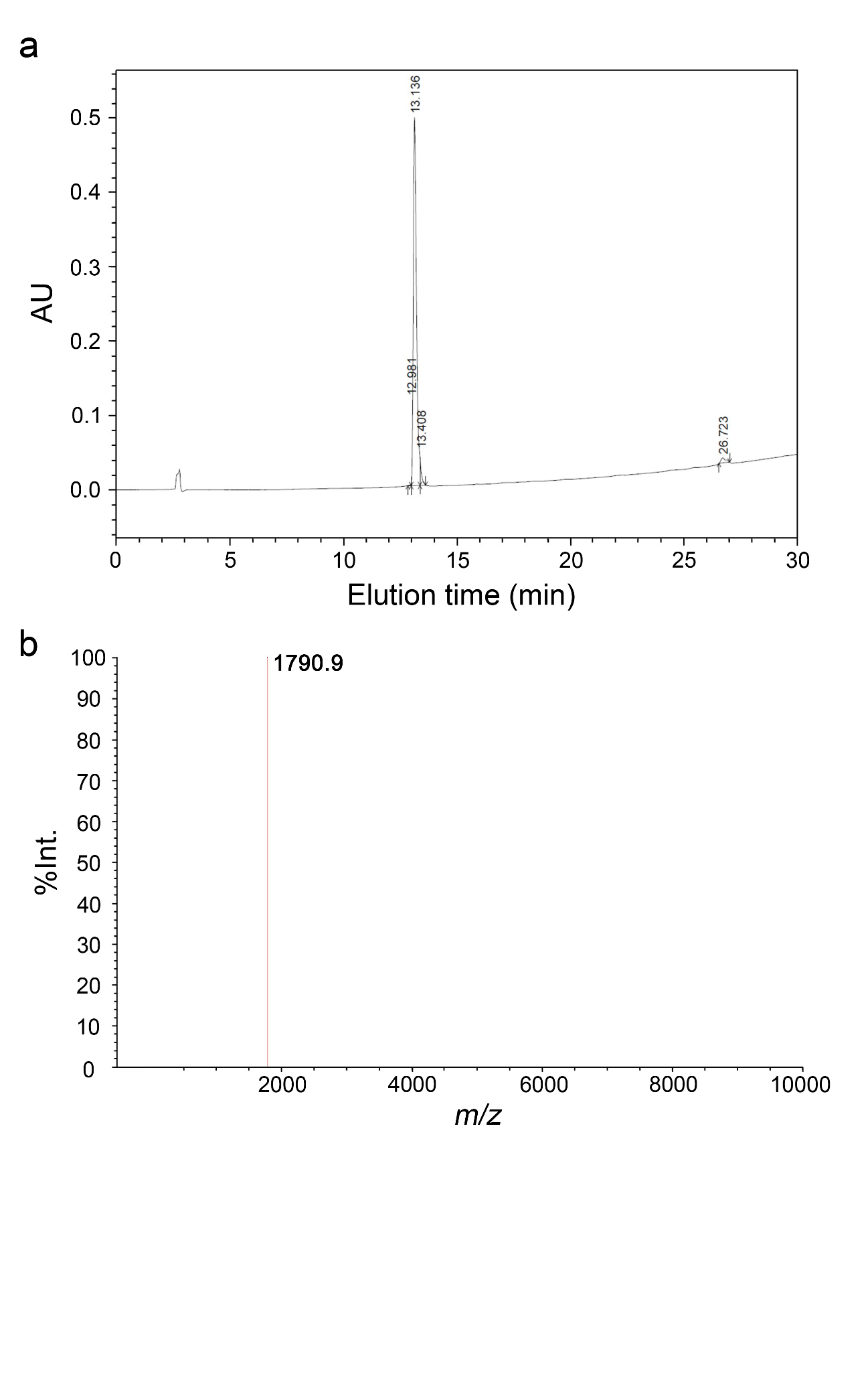
**

**Fig. S1.** (a) High-performance liquid chromatogram and (b) mass spectrum of FApep.


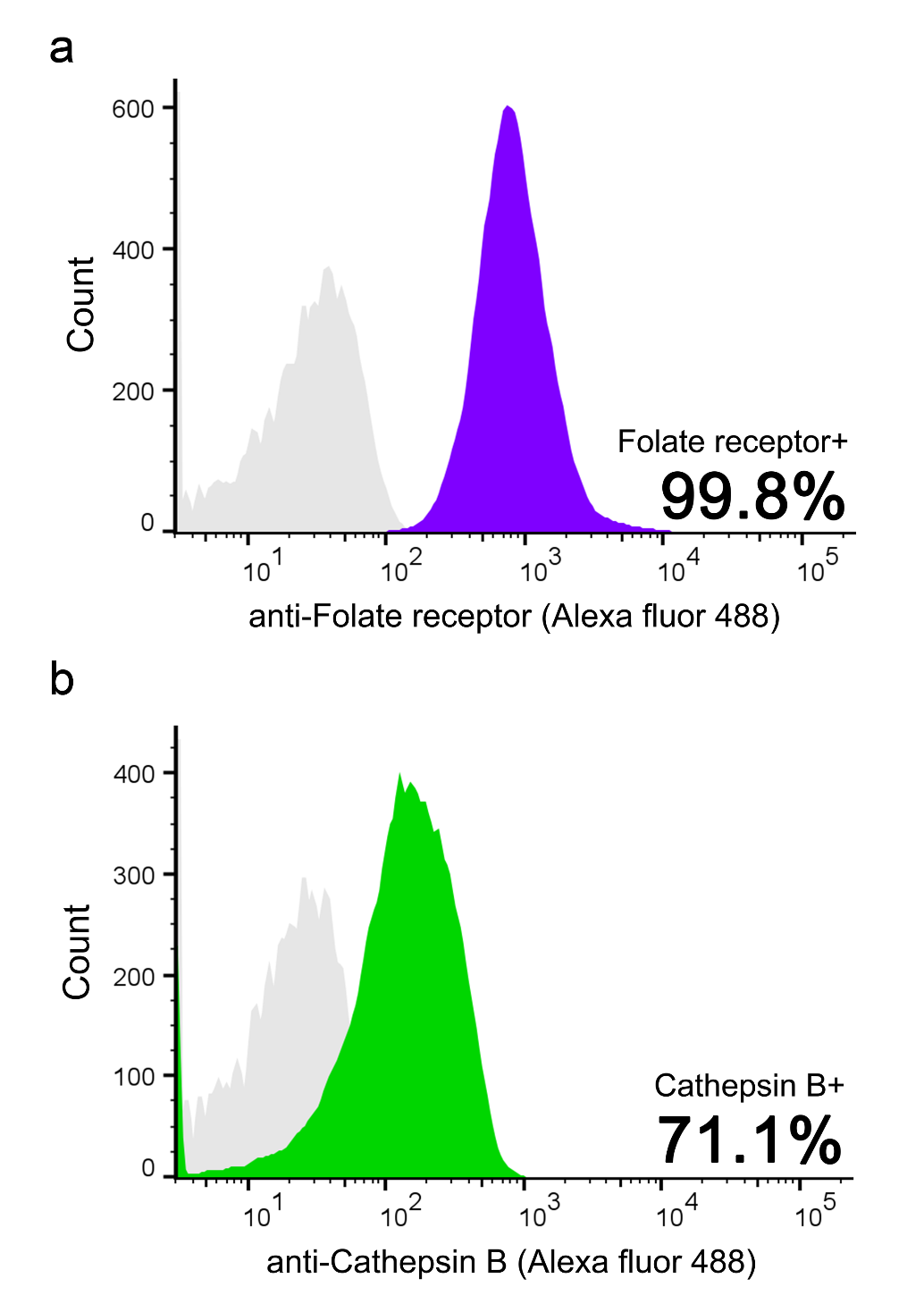


**Fig. S2.** Flow cytometry analysis of basal protein expression in LLC-1 cells. (a) Surface expression of folate receptor in LLC-1 cells. (b) Intracellular expression of cathepsin B in LLC-1 cells.

**
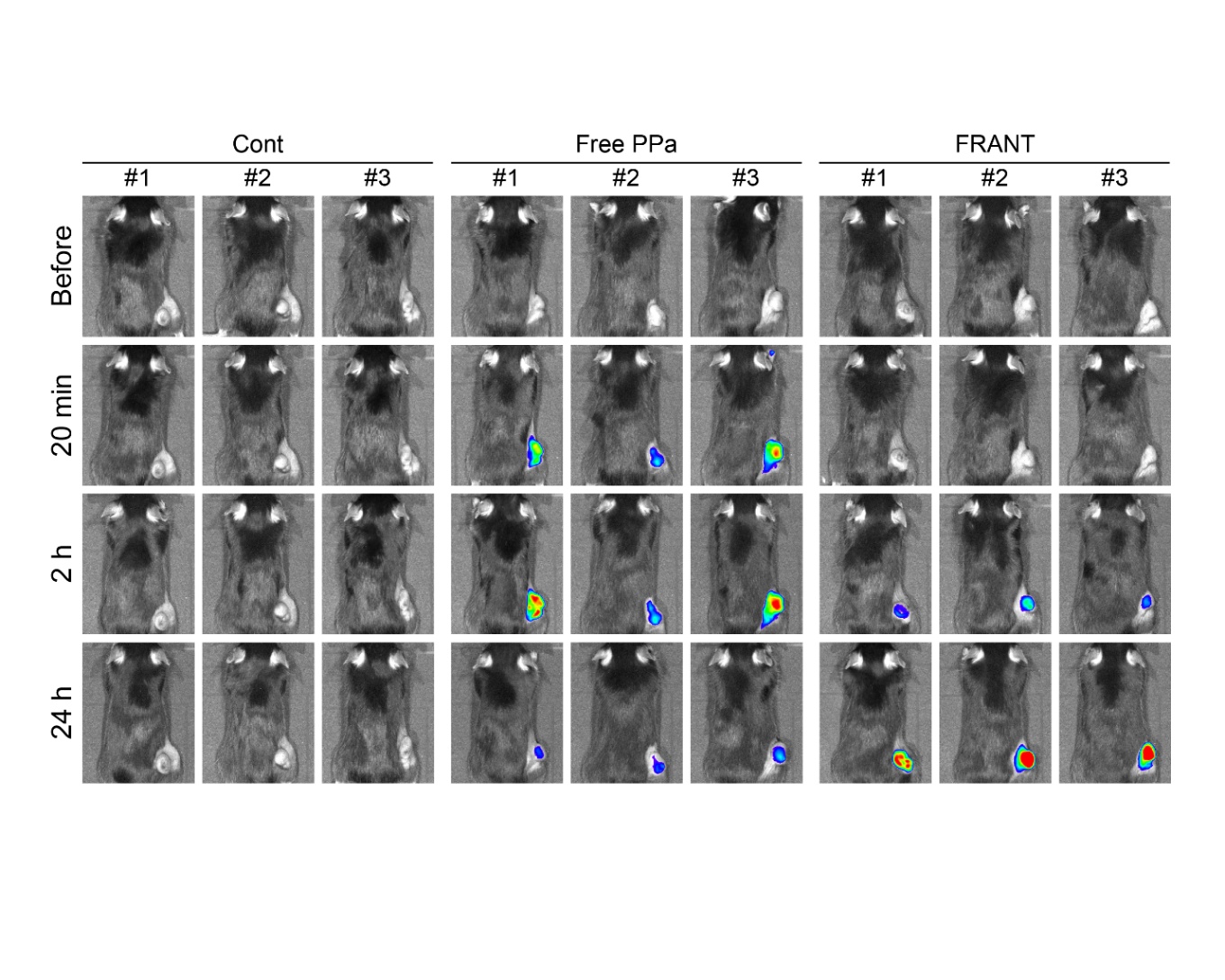
**

**Fig. S3.** *In* vivo NIR fluorescence images of LLC-1 tumor-bearing C57BL/6 mice injected with free PPa or FRANT at various time points (*n* = 3 per group).


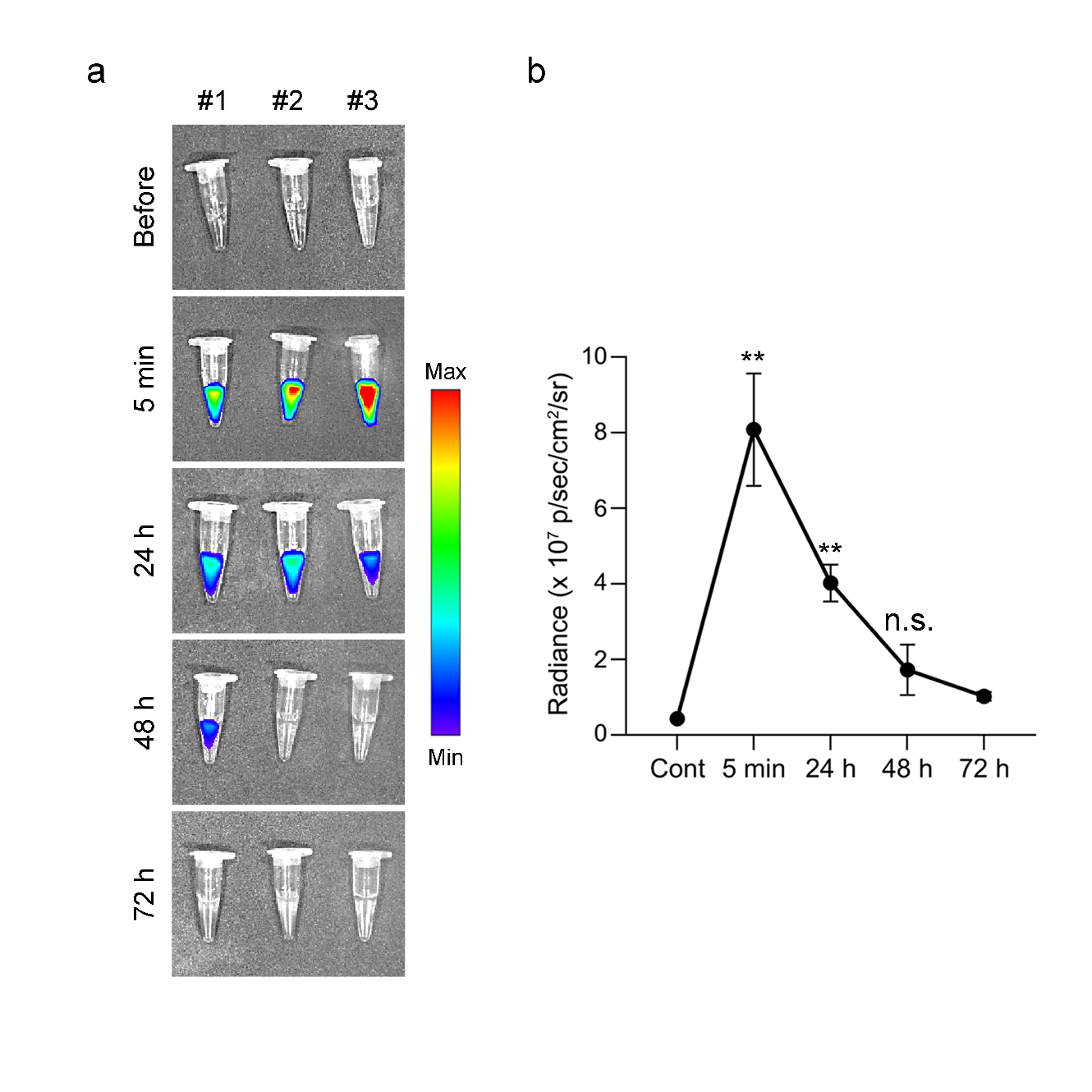


**Fig. S4.** Characterization of temporal changes in PPa fluorescence in mouse serum. *Left:* NIR fluorescence images (λ_ex._ = 620/40 nm, λ_em._ = 710/40 nm) of sample tubes containing mouse serum collected at different time points before and after FRANT injection. *Right:* Quantitative analysis of PPa fluorescence intensities derived from the NIR fluorescence images (*n* = 3).


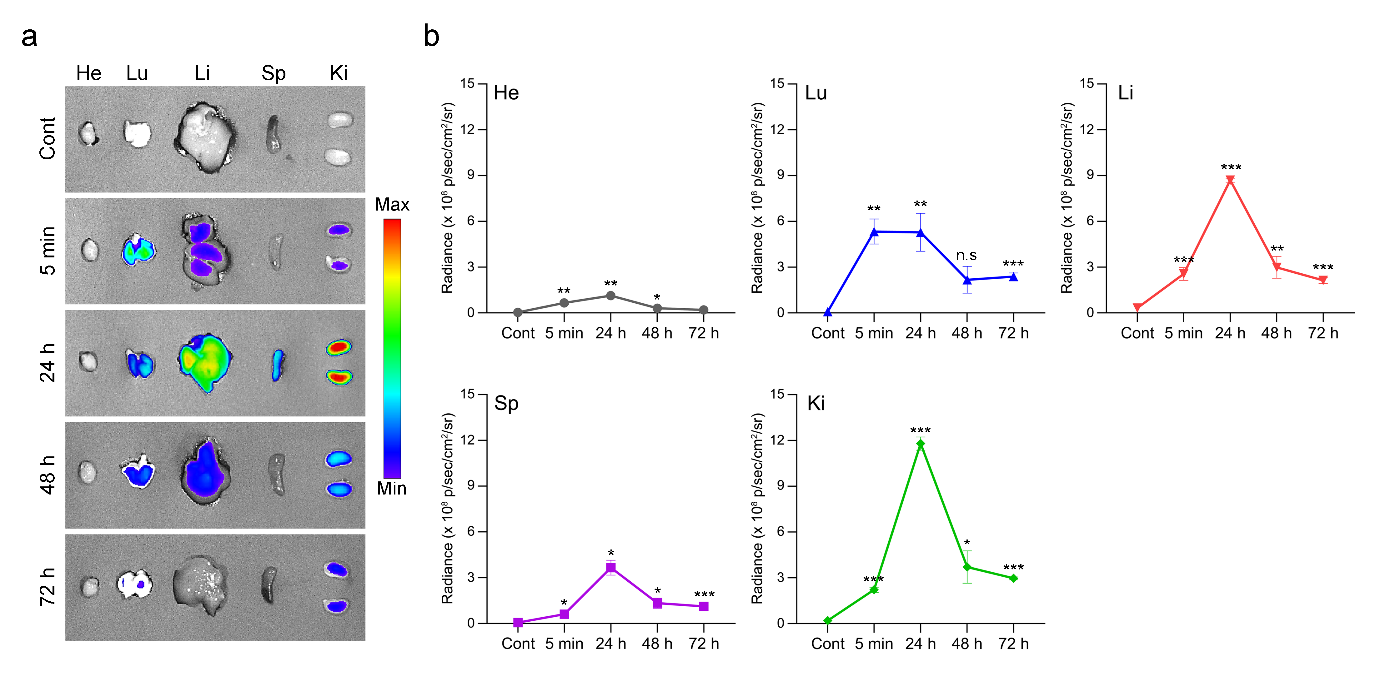


**Fig. S5.** Characterization of temporal changes in PPa fluorescence in the major organs of mice. *Left:* Ex vivo NIR fluorescence images (λ_ex._ = 620/20 nm, λ_em._ = 710/40 nm) of major organs including heart (He), lung (Lu), liver (Li), spleen (Sp), and kidneys (Ki) collected at different time points before and after FRANT injection. *Right:* Quantitative analysis of PPa fluorescence intensities derived from the NIR fluorescence images (*n* = 3).


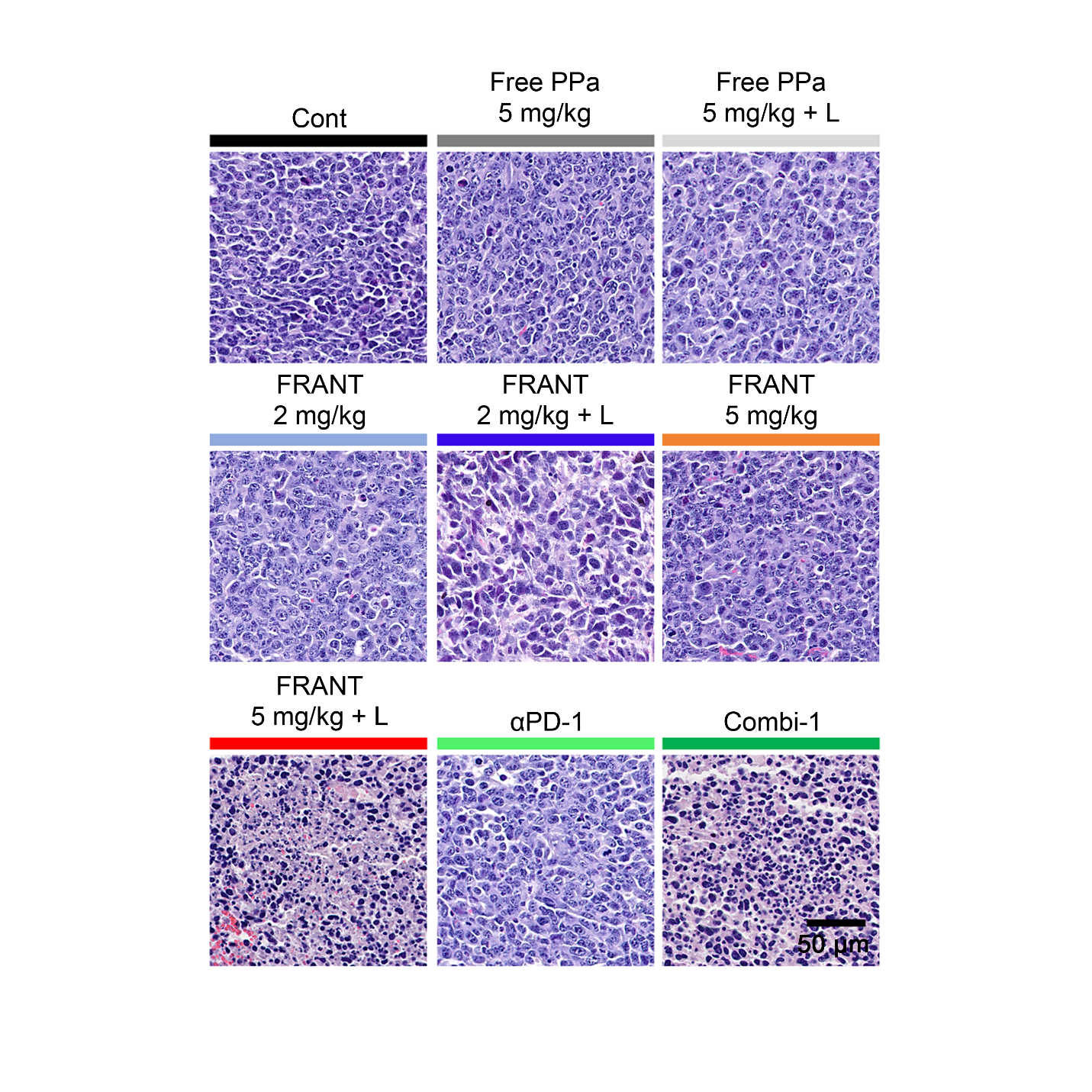


**Fig. S6.** Histopathological analysis of LLC-1 tumors. Representative H&E-stained tumor sections showing morphological changes on day 3. Scale bar, 50 μm.

**
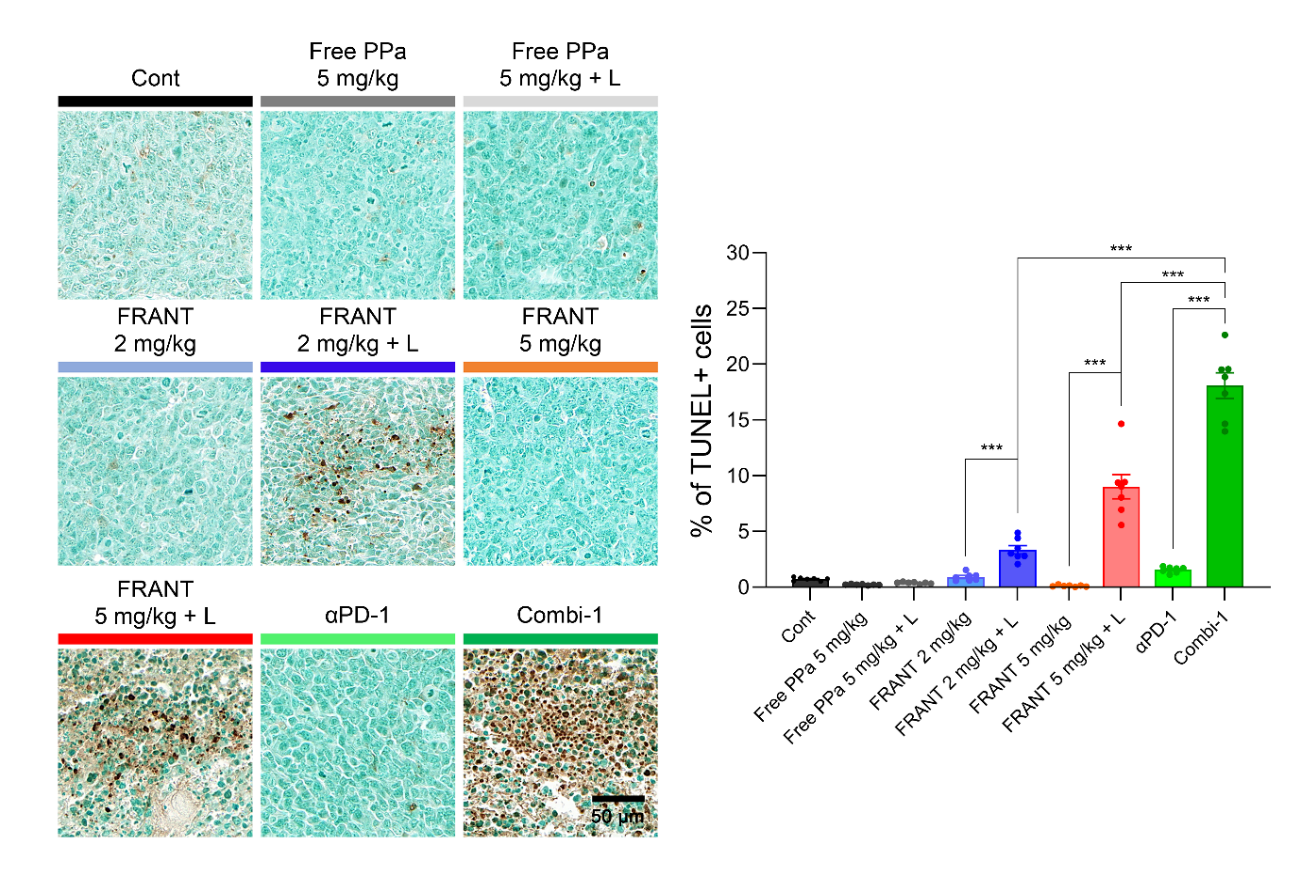
**

**Fig. S7.** TUNEL staining analysis of LLC-1 tumors. (Left) Representative images of TUNEL-stained tumor sections collected on day 3. Scale bar, 50 μm. (Right) Quantitative analysis of TUNEL^+^ cancer cells**.** Group 10 was excluded from these analyses owing to insufficient tumor size for tissue sectioning. Data are expressed as mean ± S.E. Statistical significance was determined using Student’s *t*-test (^***^*P* < 0.001).

**
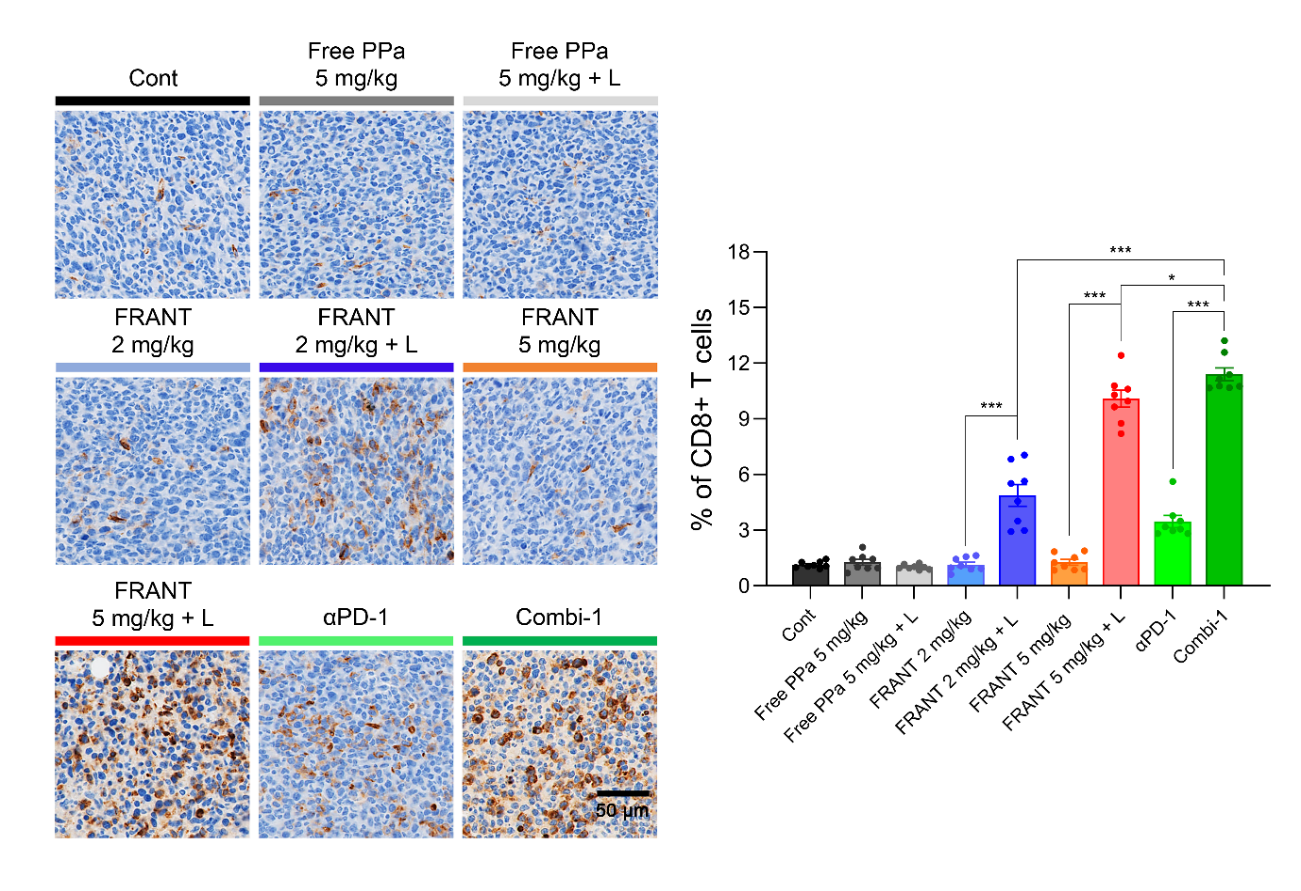
**

**Fig. S8.** Immunohistochemical analysis of CD8^+^ T cells in LLC-1 tumors. (Left) Representative IHC images of CD8^+^ T cells in tumor sections collected on day 3. Scale bar, 50 μm. (Right) Quantitative analysis of CD8^+^ T cells**.** Group 10 was excluded from these analyses owing to the insufficient tumor size for tissue sectioning. Data are expressed as mean ± S.E. Statistical significance was determined using Student’s *t*-test (^*^*P* < 0.05, ^***^*P* < 0.001).

**
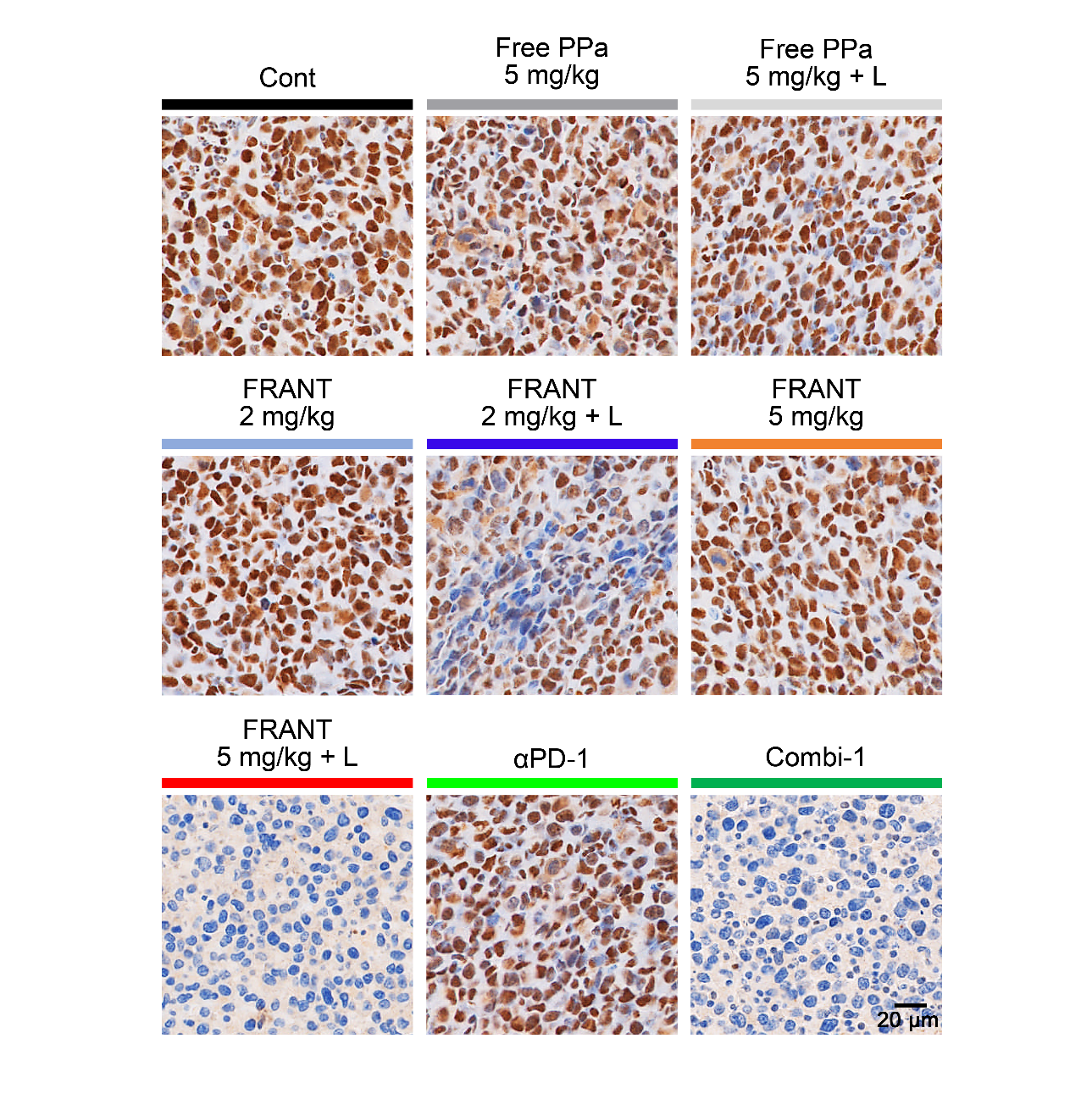
**

**Fig. S9.** Immunohistochemical analysis of HMGB1 in LLC-1 tumors. Representative IHC images of HMGB1 in tumor sections collected on day 3. Scale bar, 20 μm. Group 10 was excluded from these analyses owing to insufficient tumor size for tissue sectioning.

**
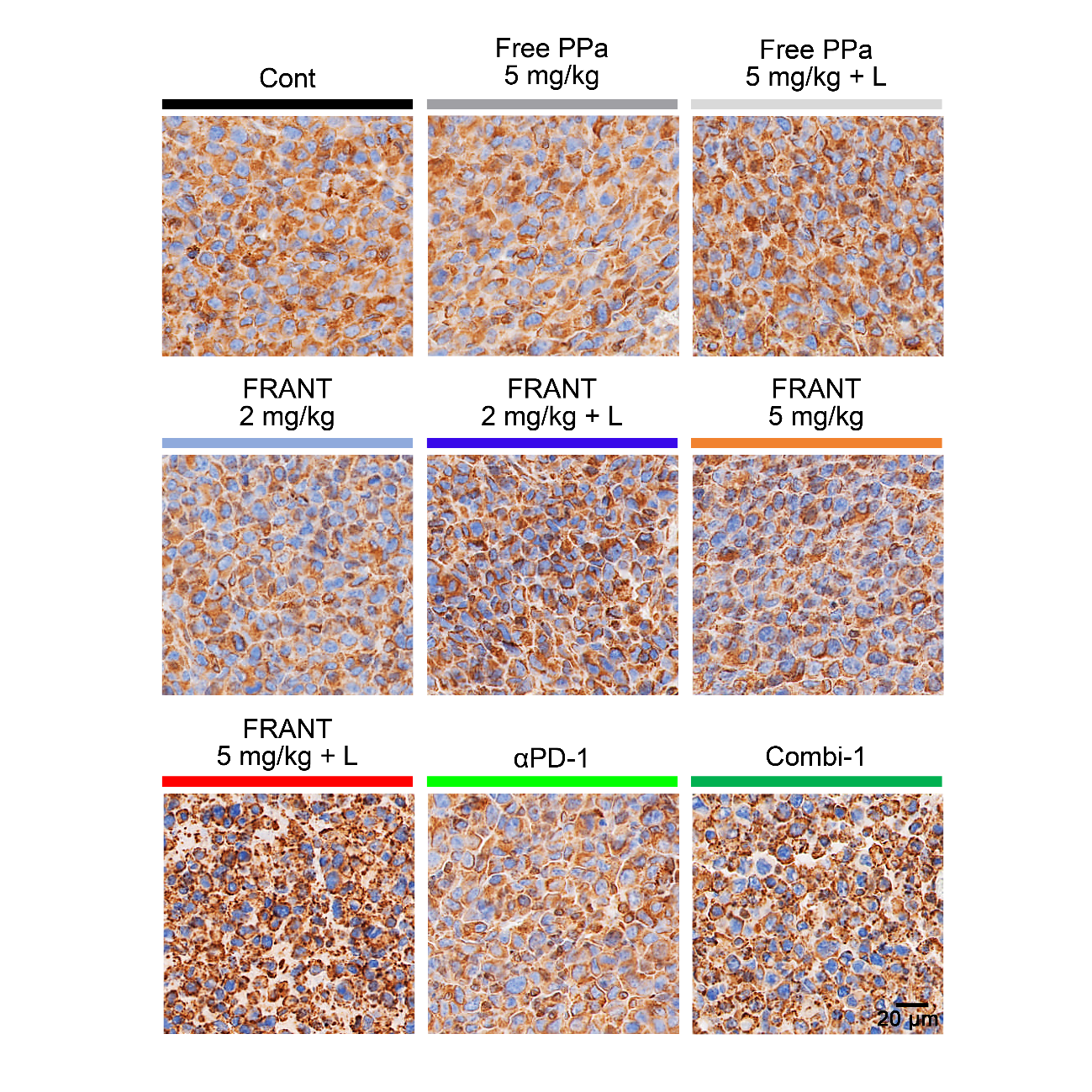
**

**Fig. S10.** Immunohistochemical analysis of calreticulin in LLC-1 tumors. Representative IHC images of calreticulin in tumor sections collected on day 3. Scale bar, 20 μm. Group 10 was excluded from these analyses owing to insufficient tumor size for tissue sectioning.

**
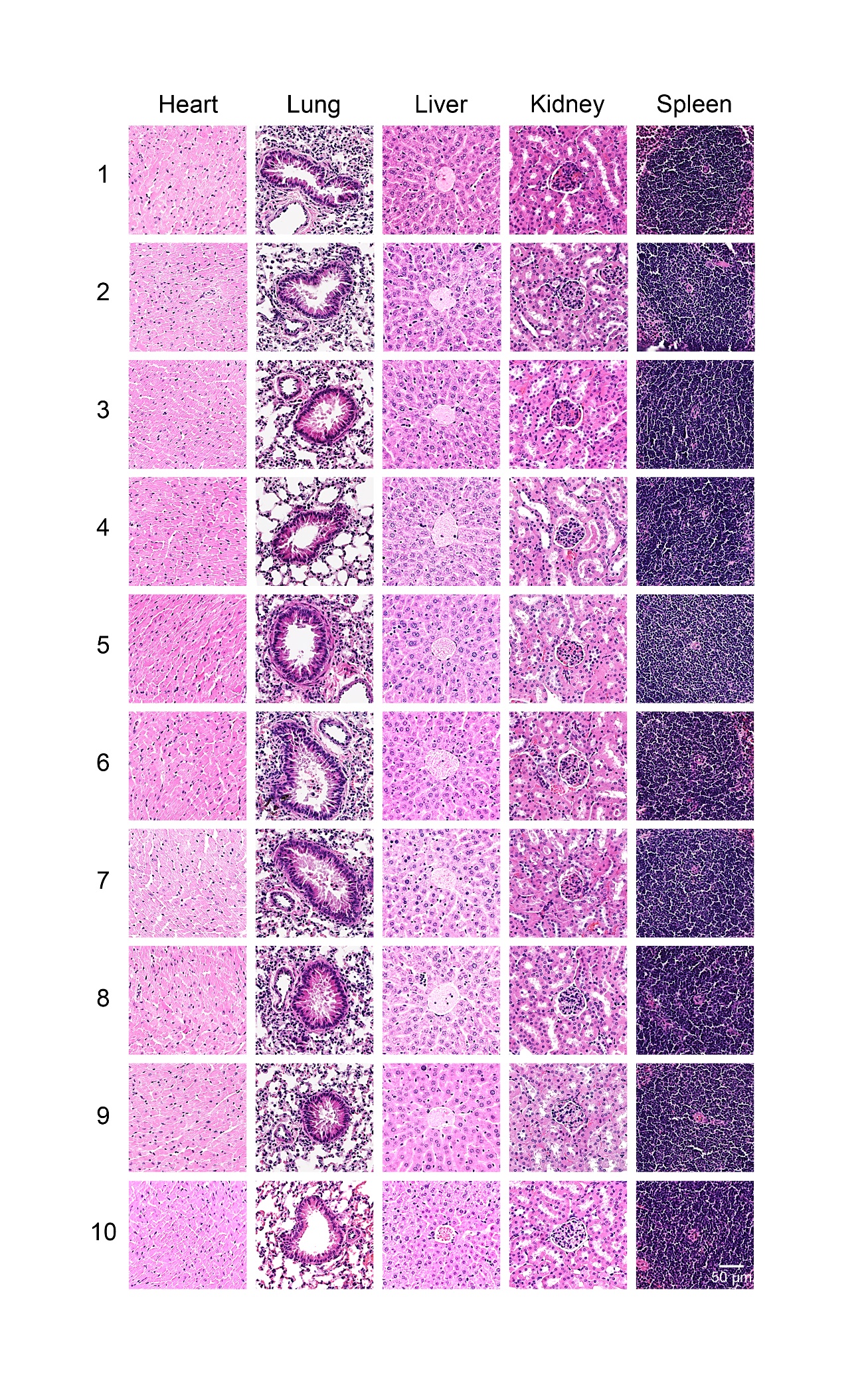
**

**Fig. S11.** *In vivo* safety of FRANT and treatment regimens. Representative H&E-stained sections of major organs from all groups (1–10) on day 10, showing no apparent histopathological changes. Scale bar, 50 μm.
